# Online Direct Infusion Mass Spectrometry of Liquid–Liquid Extraction Phases for Metabolite and Lipid Profiling with the Direct Infusion Probe

**DOI:** 10.3390/metabo14110587

**Published:** 2024-10-30

**Authors:** Cátia Marques, Lena Blaase, Ingela Lanekoff

**Affiliations:** Department of Chemistry—BMC, Uppsala University, Husargatan 3, 75 123 Uppsala, Sweden

**Keywords:** direct infusion probe, liquid–liquid extraction, glucose stimulation, INS-1 cells, time-dependent analysis, high-resolution mass spectrometry, untargeted metabolomics

## Abstract

**Background/Objectives:** Profiling of metabolites and lipids in biological samples can provide invaluable insights into life-sustaining chemical processes. The ability to detect both metabolites and lipids in the same sample can enhance these understandings and connect cellular dynamics. However, simultaneous detection of metabolites and lipids is generally hampered by chromatographic systems tailored to one molecular type. This void can be filled by direct infusion mass spectrometry (MS), where all ionizable molecules can be detected simultaneously. However, in direct infusion MS, the high chemical complexity of biological samples can introduce limitations in detectability due to matrix effects causing ionization suppression. **Methods:** Decreased sample complexity and increased detectability and molecular coverage was provided by combining our direct infusion probe (DIP) with liquid–liquid extraction (LLE) and directly sampling the different phases for direct infusion. Three commonly used LLE methods for separating lipids and metabolites were evaluated. **Results:** The butanol–methanol (BUME) method was found to be preferred since it provides high molecular coverage and have low solvent toxicity. The established BUME DIP-MS method was used as a fast and sensitive analysis tool to study chemical changes in insulin-secreting cells upon glucose stimulation. By analyzing the metabolome at distinct time points, down to 1-min apart, we found high dynamics of the intracellular metabolome. **Conclusions:** The rapid workflow with LLE DIP-MS enables higher sensitivity of phase separated metabolites and lipids. The application of BUME DIP-MS provides novel information on the dynamics of the intracellular metabolome of INS-1 during the two phases of insulin release for both metabolite and lipid classes.

## 1. Introduction

Metabolites and lipids are key factors in biology, where they are triggers, intermediates and end products of biological processes [1]. Thus, their abundancies are dynamic and provide pivotal information on the status of biological systems, including responses to stimuli [2]. Nuclear magnetic resonance (NMR) spectroscopy can be used to elucidate the chemical structure of these compounds. However, due to higher sensitivity and smaller required sample volumes, mass spectrometry (MS) is the preferred technique for identification and quantitation of lipids and metabolites in biological samples [3]. Due to the complex chemical composition of biological samples, a separation technique is usually coupled to the MS for analyte separation prior to ionization. The most commonly used online techniques for analyte separation prior to MS are liquid chromatography (LC) [4,5], gas chromatography (GC) [6,7] and capillary electrophoresis (CE) [8,9]. These have been successfully used for metabolomics and lipidomics. For example, LCMS has been used to elucidate the mechanisms behind dibutyl phthalate-promoted breast cancer [10], and aided in the discovery of biomarkers for the detection and monitoring of different stages and grades of bladder cancer [11]. However, the LC separation step for analytes prior to MS increases analysis time compared to direct infusion MS techniques [12,13,14,15,16]. Thus, there are gains to be made from establishing MS analyses using direct infusion techniques.

Several direct infusion techniques have been developed and used for metabolomics and lipidomics. The most common ionization techniques for direct infusion are matrix-assisted laser desorption ionization (MALDI) and electrospray ionization (ESI) MS [17]. While MALDI MS is always based on laser ablation of a solid sample, there are several approaches for sample introduction for ESI MS. These include directly infusing the sample using a syringe pump [18]; introducing the sample via a sample loop into a flowing solvent stream (flow injection analysis (FIA)) [19,20,21,22]; and self-aspirating the sample through the ESI emitter using the Venturi effect [23,24,25,26,27]. One system that uses the Venturi effect is our previously reported direct infusion probe (DIP) coupled to MS [28]. The DIP enables samples to be rapidly switched with minimal carryover while avoiding the longitudinal diffusion characteristic of FIA [28]. Nevertheless, a challenge with all direct infusion MS systems is the inherent ionization suppression that can limit analyte ionization efficiency and therefore detectability in chemically complex samples.

The chemical complexity can be reduced by adding a sample clean-up step, such as solid phase extraction (SPE) or liquid–liquid extraction (LLE), prior to analysis [29]. In LLE for lipidomics and metabolomics of biological samples, one major goal is to isolate lipids and metabolites in different phases, as first described in the 1950s by Folch et al. [30] and Bligh and Dyer (BD) [31]. In these methods, a mixture of methanol and chloroform or dichloromethane generates two phases where lipids primarily partition into the organic-rich phase. For example, the BD method has been used to ascertain a conserved lipid profile in mutant *KRAS* lung cancer (KMLC) patient-derived xenografts compared to primary patient specimens [32]. Furthermore, BD was employed to show that acute radiation exposure causes alterations in lipid metabolism correlated with cardiac dysfunction [33]. However, despite the high performance of BD, both chloroform and dichloromethane are highly toxic and carcinogenic; thus, other LLE systems have been explored and developed to obtain more sustainable LLE methods [29].

Two other LLE methods are the butanol–methanol (BUME) method and the three-phase liquid extraction (3-PLE) method. The BUME method was first reported by Löfgren et al. and includes a mixture of butanol, methanol, hexane, ethyl acetate and water that forms the organic and aqueous-rich phases [34]. For example, the BUME method was used in a study showing that diets rich in saturated fatty acids promote fat accumulation and increase serum ceramides, while the opposite was found for polyunsaturated fatty acid-rich diets in overweight humans [35]. The 3-PLE method was established by Vale et al. [36,37]. In this method, the mixture of hexane, methyl acetate, acetonitrile and water generates a three-phase system that has two organic-rich phases and one aqueous-rich phase. The 3-PLE method was used in a study confirming that a fatty acid synthase inhibitor induces oxidation of the polar lipid fraction of KMLC cells without influencing neutral lipids [32]. Another study showed tissue-specific and temporal alterations in lipid homeostasis and reported that more than one time point should be considered when studying lipid homeostasis in metabolic diseases [38]. Overall, various LLE methods can be used in a wide range of applications to learn about the chemistry behind biological function and dysfunction.

Here, we present the online combination of LLE with DIP-MS to reduce the chemical complexity of the samples while maintaining rapid direct infusion MS analysis. By simply dipping the probe into the respective phase, we report rapid analysis of the respective phases for metabolomics and lipidomics of biological samples. We compared the three LLE methods, BD [39], BUME [34] and 3-PLE [36], for compatibility with online LLE DIP-MS using a tissue extract. Following, the BUME method was selected for further characterization and utilization in a study showing intracellular time-resolved chemical dynamics of INS-1 cells upon exposure to glucose. Overall, we report a successful and rapid pre-separation of the organic and aqueous-rich phases of both lipids and metabolites in chemically complex biological samples using LLE and direct infusion analysis.

## 2. Materials and Methods

Chemicals: The solvents used were analysis-grade formic acid (Merck, Darmstadt, Germany), GC grade 2-butanol (Merck, Darmstadt, Germany), LC-MS-grade acetonitrile (Thermo Fisher Scientific, Waltham, MA, USA), HPLC-grade heptane (Thermo Fisher Scientific, Waltham, MA, USA), LC-MS-grade methanol (Honeywell, Charlotte, NC, USA), LC-MS-grade ethyl acetate (Honeywell, Charlotte, NC, USA), technical-grade hexane (VWR, Radnor, PA, USA), HPLC-grade chloroform (Acros Organics, Waltham, MA, USA) and Milli-Q water from Milli-Q Plus.

The Standards used were L-alanine (≥99.5%), L-arginine (98.5–101.0%), L-asparagine (≥98%), L-aspartic acid (≥99%), L-cysteine (≥98.5%), D-glutamine (≥98%), L-glutamic acid (98.5–100.5%), glycine (98.5–101.0%), L-histidine (≥99%), L-isoleucine (≥98%), L-leucine (≥98%), L-lysine (≥98.0%), L-methionine (99.0–101.0%), L-phenylalanine (99%), L-proline (≥99.5%), L-serine (≥99%), L-threonine (≥98%), DL-tryptophan, L-tyrosine (purissimum-grade), L-valine (≥99.5%), γ-aminobutyric acid (≥99%), O-acetyl-L carnitine hydrochloride (≥99%), choline chloride (≥99%), creatine monohydrate (99%), Cell-Free Amino Acid Mixture—15N (98%), glucose-d_2_, γ-aminobutyricacid-d_2_ (GABA-d_2_), acetylcholine-d_9_, oleic acid-d_9_ (FA 18:1-d_9_) and LPC 19:0, PC 11:0/11:0. The concentrations of labeled and non-labeled standards used for the different experiments can be found in Tables S1 and S2. The IS solutions were prepared in Milli-Q water for the liquid–liquid extractions or in MeOH:H_2_O (9:1) + 0.1% formic acid.

Liquid–liquid extraction protocols: The different extractions were performed as previously described. Briefly, for the BD [39], 80 µL of chloroform, 80 µL of methanol and 72 µL of water (containing the sample) were mixed and vortexed for 10 s. For the BUME extraction [34], 30 µL of the sample were mixed with 100 µL of butanol/methanol (3/1), vortexed for 5 s and then 50 µL of heptane/ethyl acetate (3/1) and 100 µL 1% formic acid were added and the solution was vortexed again for 10 s. For the 3-PLE method [37], 80 µL of hexane, 80 µL of methyl acetate, 60 µL of acetonitrile and 80 µL of water (containing the sample) were mixed and vortexed for about 10 s. After allowing the phases to separate for a couple of minutes, they were analyzed using online LLE DIP-MS.

BUME characterization: The solvents were mixed according to the BUME protocol [34]. Following, each phase was separated and solutions containing known concentrations of analytes were prepared in each phase at similar concentrations and analyzed with DIP-MS to generate response curves (Tables S1 and S2).

Biological samples: Intact frozen rat brain tissue (bought from Innovative Research Inc., Novi, MI, USA) was chopped into small pieces and 35 mL of MeOH was added. The sample was sonicated using an ultrasonic processor (VCX 130, Sonics and Materials, Inc., Newton, CT, USA) for 2 cycles at 50% for a total of 5 min, pulsed for 2 s with 1 s pauses in between the cycles in order to achieve cell dissociation. Finally, the sample was centrifuged at 2000× *g* for 6 min and the supernatant was collected and stored at −20 °C until use. The final extract contained 34.61 mg of rat brain tissue per mL.

Rat insulinoma cell line INS-1 clone 832/13 cells were maintained in RPMI 1640 (Invitrogen, Walthan, MA, USA) containing 10 mM glucose and supplemented with 10% Newborn Calf Serum, L-glutamine (2 mM), penicillin (100 μg/mL), streptomycin (100 μg/mL), sodium pyruvate (1 mM) and β-mercaptoethanol (50 μM). The cells were kept at 37 °C in a humid atmosphere containing 5% CO_2_. Cells were counted and grown in 24-well plates. After 24 h, the cells were washed with 1× PBS and incubated with 3 mM glucose for 1 h at 37 °C without CO_2_. Afterward, the cells were exposed to 20 mM extracellular buffer containing 138 mM NaCl, 5.6 mM KCl, 1.2 mM MgCl_2_ and 2.6 mM CaCl_2_ at pH 7.4 for ten different durations at 37 °C without CO_2_. Three wells were prepared for each glucose exposure time: 0, 1, 2, 3, 4, 5, 6, 8, 10 and 15 min. After, the cells were quickly washed with water, and analytes were extracted by adding 150 µL of butanol/methanol (3:1) and sonicating for 2 min. A 100 µL volume of this extract was stored at −80 °C. From the cell extract, a volume equivalent to 27,000 cells (experimental section of Supplementary Materials) was pipetted and freeze-dried and dissolved in different solvents: for MeOH:H_2_O (9:1) DIP-MS analysis, 54.1 µL of MeOH:H_2_O (9:1) with 0.1% formic acid was added and the samples were sonicated for 2 min. For BUME extraction, 40 µL of butanol/methanol (3:1) was added, followed by 12 µL of water. The sample was then sonicated for 2 min. Subsequently, 20 µL of heptane/ethyl acetate was added, followed by 40 µL of 1% formic acid. The solution was then vortexed for 10 s. All samples were kept at −80 °C until analysis.

Mass spectrometric parameters: An Orbitrap™ IQ-X™ Tribrid™ (Thermo Fisher Scientific, Bremen, Germany) was used to acquire data in positive and negative modes in full scan mode using a mass resolution of 240k (m/Δm at m/z 200) and a transfer tube temperature of 275 °C. The applied high voltage was optimized for each solvent (Table S3).

Direct infusion probe (DIP): The DIP-MS system was set up as described in Marques et al. [28]. Briefly, a 7 cm stainless-steel capillary (320:50 µm OD:ID) (MS Ekspert Sp. z o.o., Gdańsk, Poland) was inserted in a PEEK sample tee (0.050″ through hole) (Upchurch Scientific, Oak Harbor, WA, USA) with Teflon sleeves with 1/16-inch OD and 0.5 mm ID (VICI Valco, Houston, TX, USA). The probe was held in place using a 3D-printed holder attached to the nano-spray interface of the MS. The sample vial was positioned manually and held in place using a MIM micro-manipulator (Quarter Research and Development, Bend, OR, USA).

Data analysis: The collected data were extracted with the software MZmine 2.53 [40] according to the details in the experimental section of the supporting information. Graphs were generated using in-house RStudio 2023.03.0 scripts [41].

## 3. Results and Discussion

### 3.1. Online LLE DIP-MS

Direct infusion MS provides rapid simultaneous detection of all ionizable compounds in a sample. Consequently, a complex chemical matrix, including salts, proteins or endogenous metabolites and lipids, can suppress analyte signals and limit analyte detectability [42,43,44,45]. Although these effects can be minimized by sample preparation, such as LLE or SPE, sample preparation is typically laborious, thus it adds time to each analysis and can be a source of errors, including analyte degradation and losses. Here, we report the combination of LLE with DIP-MS, where we directly sample the LLE phases with the DIP. This enables rapid online sampling and increased detection of analytes partitioned into the different phases without the added time required for pre-separation of the phases.

**Figure 1:**
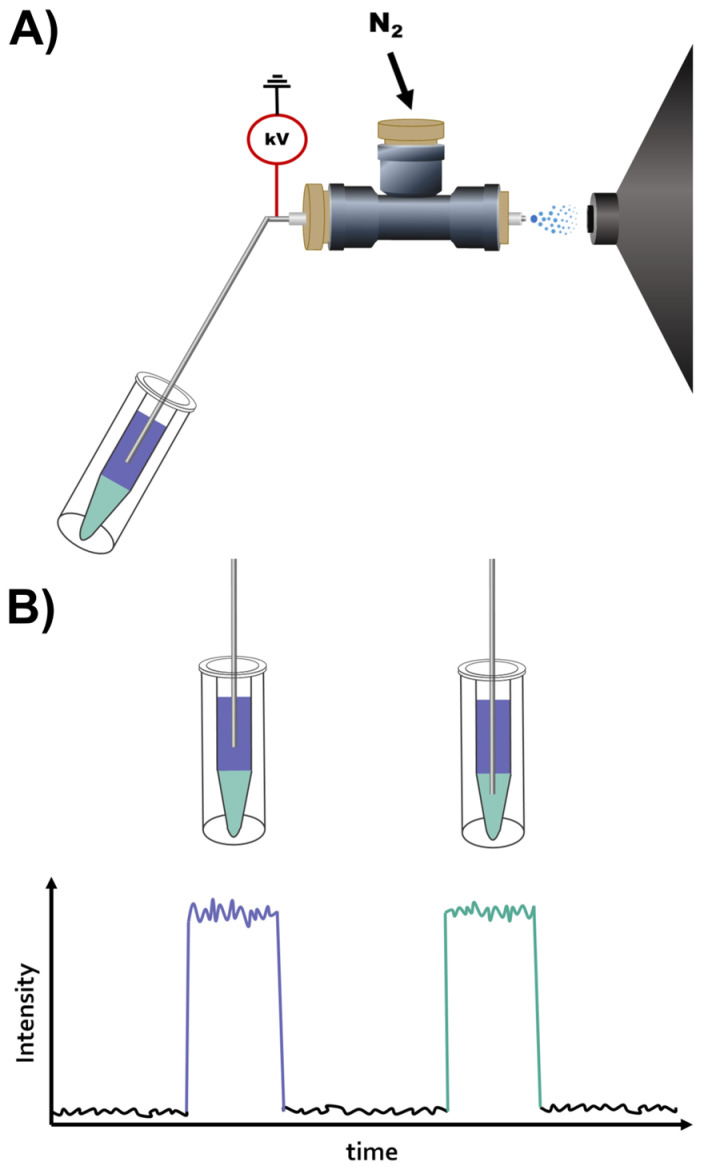
(**A**) Schematic of online LLE DIP-MS sampling with two phases depicted in purple and green. (**B**) Schematic of sampling and data from the upper and lower LLE phases of online LLE DIP-MS.

The procedure for this analysis is trivial and only includes two steps. Firstly, the sample is placed into a vial followed by the addition of the solvents for LLE and the equilibration of the phases (Figure 1A,B). Secondly, the vial is positioned by the instrument for DIP-MS analysis. The vial is positioned so that the tip of the DIP is submerged in the respective phase to be analyzed. For example, the tip can be first positioned in the upper phase for 40 s, of which 10 s are used to equilibrate the capillary to the new sample, followed by 30 s of data acquisition in one phase. This will ensure that the signal is stable and that there is no carryover between the phases, including the interface of the phases [28]. Subsequently, the DIP can be repositioned to the lower phase for a repeat of the measurement procedure. After these two steps, the acquired data are ready to be processed and evaluated. This simple workflow provides an efficient way to analyze both lipids and metabolites in their respective phases with DIP-MS with reduced matrix effects and without the need for time-consuming manual phase separations and potential losses when transferring material between containers.

### 3.2. Comparison of Liquid–Liquid Extraction Methods

The major reason for performing LLE is to reduce the chemical complexity that may cause interferences during the ionization of chemically complex biological samples by separating lipids and polar metabolites. Here, the applicability of three different LLE methods, BD [39], BUME [34] and 3-PLE [37], for online LLE DIP-MS was evaluated. The three LLE methods were selected since they had similar reported efficiencies in separating lipids and metabolites despite their vastly different solvent systems [34,37]. For comparison, a methanolic extract of rat brain was used; this enabled direct evaluation of analyte phase separation and detectability despite the high chemical complexity. Prior to analysis, the tissue extract and the solvents for the respective LLE methods were added to silanized glass vials. Upon equilibration of the respective LLE phases, the phases were analyzed by inserting the DIP probe into each phase and acquiring data in both positive and negative modes. To ensure good spray stability and analyte detectability in all analyses, the voltage settings for ionization were optimized for each phase (Table S3). The ESI of all phases was found to be stable with a TIC variation of below 20%, except for the middle organic and lower aqueous phases of 3-PLE, where the TIC variation was up to 30%. Thus, despite using these unconventional organic solvents for ESI, the LLE DIP-MS setup was found to be robust.

**Figure 2:**
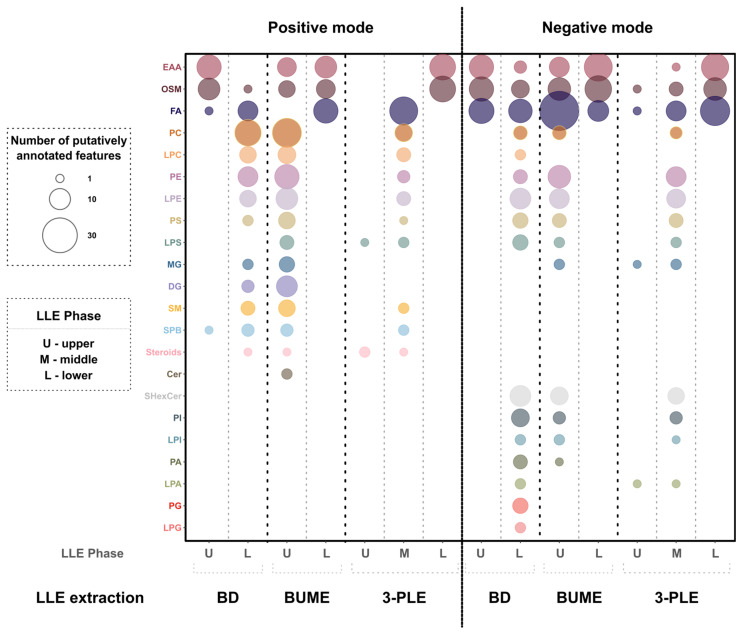
Analysis of tissue extract with online LLE DIP-MS from individual phases of three LLE methods (BD, BUME, and 3-PLE) in positive (left) and negative (right) modes. Bubble size represents the number of putatively annotated analytes for each class using an in-house-generated analyte list. The abbreviations of the classes are as follows: EAA, essential amino acids; OSM, other small metabolites; FA, fatty acyls, fatty acids, and fatty aldehydes; PC, phosphatidylcholines; LPC, lysophosphatidylcholines; PE, phosphatidylethanolamines; LPE, lysophosphatidylethanolamines; PS, phosphatidylserines; LPS, lipopolysaccharides; MG, monoacylglycerols; DG, diacylglycerols; SM, sphingomyelins; SPB, sphingoid bases; Steroids, sterols and sterol lipids; Cer, ceramides; SHexCer, sulfatide species; PI, phosphatidylinositols; LPI, lysophosphatidylinositols; PA, phosphatidic acids; LPA, lysophosphatidic acids; PG, phosphatidylglycerols; and LPG, lysophosphatidylglycerols.

To compare detected analytes from the extract in the different methods and phases, all data were aligned, and the detected metabolites and lipids were putatively annotated (experimental section of Supplementary Materials). Following, lipids and metabolites were grouped according to their molecular classes and plots were generated, with the circle size corresponding to the number of annotated molecules in that group (Figure 2, Tables S4 and S5). The results show a great difference in detected analytes between the LLE methods, LLE phases, and ionization modes. Overall, all LLE methods were found to provide a wide coverage of detected classes, despite the use of unconventional ESI solvents. Moreover, in all LLE methods, small polar metabolites were separated from phospholipids and neutral lipids, which is expected based on previous work [34,36,46]. For BD, the lower organic-rich phase contained a large amount of different lipid classes in both positive and negative ionization modes. Similarly, the upper organic-rich phase of BUME contained a large amount of lipid classes. In 3-PLE, the majority of the lipids in the negative mode were detected in the middle organic-rich phase while in the positive mode, both the middle and the upper organic-rich phases contained lipid species. Overall, the partitioning of the compounds between the aqueous and organic phases was found to be in agreement with their log *p* values (Table S6). The compounds with log *p* < 1, which are small metabolites, were found in the aqueous phases, while compounds with log *p* > 1, corresponding to lipids, were found in the organic-rich phases [47]. Comparably, the BD and BUME methods provided a higher coverage of lipid species than the 3-PLE method. For instance, in positive mode, BD and BUME enabled the detection of 62 and 90 lipid species, respectively, while 3-PLE enabled the detection of 48 lipid species when considering both organic phases (Tables S4 and S5). The BUME method enabled the detection of more lipid species than the BD method, with 90 and 85 species detected for BUME and 62 and 75 species detected for BD in the positive and negative modes, respectively. However, the BD method contained a large amount of the toxic solvent chloroform in the lower phase of BD, which should be avoided if possible. Thus, out of the three evaluated methods, the preferred method for online LLE DIP-MS was found to be BUME.

### 3.3. Characterization of BUME for Online LLE ESI-MS

**Figure 3:**
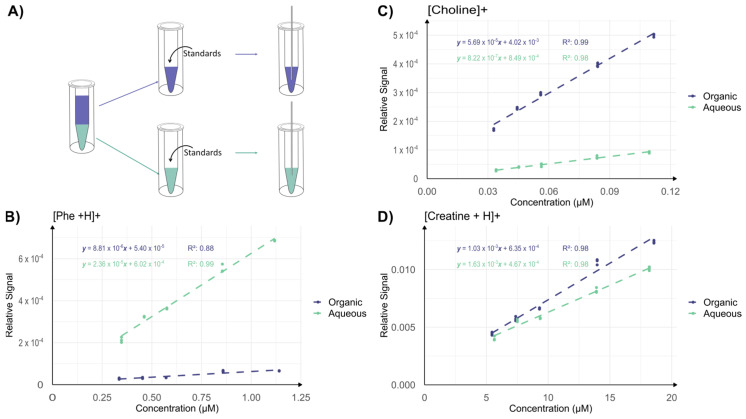
DIP-MS analysis of pre-separated and standard doped BUME phases. (**A**) Experimental setup: the phases used in the BUME extraction were separated and standards were added to each phase, which were then analyzed with DIP-MS. (**B**–**D**) Relative signals (normalized to TIC) for standards at different concentrations. In each graph, the data for the lower aqueous phase are presented in green and for the upper organic phase in purple. *n* = 3 for each point.

In BUME, the upper organic-rich phase mainly contains lipids, while the lower aqueous-rich phase mainly contains metabolites. However, artifacts during ionization of either phase may limit detectability. In an experiment, a larger volume of BUME solvents was mixed and the two phases were manually separated into two vials of equal volume (Figure 3A). Following, equal volumes of a standard solution containing known metabolites and lipids at known concentrations were added into each vial containing either the organic or the aqueous BUME phase, for subsequent DIP-MS analysis (Figure 3A, Tables S1 and S2). The acquired data were plotted as response curves, with the signal of the analyte normalized to the total ion current (TIC) (Figure 3B–D and Figure S1). The TIC is on average 8 × 10^7^ for the aqueous phase and 2 × 10 ^8^ for the organic phase. Several observations can be made: the slope of phenylalanine is much higher in the aqueous phase (2 × 10^−5^) compared to the organic phase (9 × 10^−6^), the slope of choline is much higher in the organic phase (6 × 10^−5^) than in the aqueous phase (8 × 10^−7^) and, the slope of creatine is similar in the organic (1 × 10^−3^) and aqueous phases (1.6 × 10^−3^). This suggests that analytes can be ionized in both phases, but the efficiency of ionization of each species is solvent-dependent. The ion evaporation model [48,49] of ionization mechanisms in ESI states that small analytes close to the droplet surface will preferentially be ejected and thereby ionized [42,48,50,51,52]. Thus, in addition to differences in the evaporation of the solvent, our results indicate a solvent-dependent difference in the distribution of choline, phenylalanine and creatine inside the ESI droplets that directly impacts ionization. Nevertheless, these results show that both the aqueous-rich and the organic-rich phases provide reasonable but analyte-specific ESI of metabolites and lipids.

### 3.4. Rapid Workflow for Cellular Metabolomics and Lipidomics

Metabolite and lipid profiling with online LLE DIP-MS opens up new possibilities to rapidly analyze a larger number of low-volume cellular extracts with low cell density to learn about chemical alterations in biological systems. The established workflow was therefore used to analyze the many samples required for time-resolved analysis of metabolic alterations in insulin-secreting cells after glucose exposure. In this experiment, INS-1 cells were cultured to confluency in 24-well plates, averaging 405,750 cells/well, with an RSD of 6% as determined by manual counting using a Bürker chamber. Following, the attached cells were quickly washed with water to remove media contaminants and then butanol/methanol (3:1) was added to lyse the cells and extract analytes. An aliquot of the extract, corresponding to 27,000 cells, was used for LLE DIP-MS analysis. This low amount of cells is orders of magnitude less than typical omics studies [53,54]. In addition to BUME DIP-MS, an equal aliquot of the sample was analyzed using the one-phase solvent, methanol/water (9:1). This was chosen for comparison of detected analytes from the cells since previous reports have used solvent systems containing different compositions of methanol/water to detect small metabolites and lipids with direct infusion techniques [28,55,56,57,58,59]. For the LLE samples, the final cell density was 242 cells/µL of total volume or, in the case of full partitioning, 483 cells/µL and 520 cells/µL for aqueous and organic phases, respectively. For the MeOH:H_2_O solution, the final cell density was 500 cells/µL, which is an almost equal amount of cells for comparison between the methods. Furthermore, the volume of each LLE phase and the methanolic solution was the same, 54 µL, showcasing that the LLE DIP-MS can be used to analyze samples containing low cell numbers in small volumes.

The sample aliquots were analyzed with DIP-MS, either the one-phase MeOH:H_2_O or the two BUME phases, and the detected analytes were putatively assigned using accurate mass (<5 ppm). The results show that the BUME DIP-MS increased the detectability of lipids compared to MeOH:H_2_O (Figures S2–S4). In particular, the organic-rich phase of BUME enabled the detection of additional lipid classes, which showed intensities >1.3 times the blank in all three replicates in both positive and negative modes. For example, FA, MG, PI, PS, PG, SPB and n-acyl taurine species were only detected in the organic-rich phase of BUME (Figures S2–S4). In the aqueous-rich BUME phase, the detected metabolites were similar to the metabolites detected in the methanolic solvent, including amino acids, creatine, taurine and glucose derivatives (Figures S3 and S4, Tables S7 and S8). However, in our experience, the methanolic solvent in general provided better ionization than the aqueous phase. The quick separation step of lipids and metabolites into two phases, in the same vial used for analysis, was achieved within 45 s per phase. This includes time for sample movement and acquisition of 30 s of stable data, making this method ideal when higher throughput analysis is required. Overall, this rapid workflow with minimal manual handling time provides opportunities to increase the number of detected analytes per sample, even samples with low volumes and low cell densities, and allows for analysis of a large number of samples within minimal time.

### 3.5. Time-Resolved Analysis of Glucose-Exposed INS-1 Cells

One biological system that is chemically dynamic over time is the release of insulin from beta cells upon exposure to glucose. The immortalized INS-1 cell line enables the study of the insulin mechanism behind type 2 diabetes [60]. Specifically, upon an increase in glucose in the medium, the cellular metabolome is altered for the release of insulin. This process has been shown to occur in a biphasic manner in individuals, where the first phase starts within 2 min and lasts for around 10 min, and plateaus 2–3 h after glucose exposure [61]. The intricate internal events that occur within the first 15 min of glucose exposure are still being elucidated [61]. With the fast, sensitive and high-throughput analysis of minute samples that is achievable with online LLE DIP MS, the metabolome dynamics in INS-1 cells upon glucose exposure can be studied at several time points.

The metabolome dynamics at different time points can potentially be further clarified with the less complex chemical matrix by using LLE-DIP MS. In an experiment, INS-1 cells were cultured to confluency in 24-well plates, as described above. Following, the cells in all the wells were exposed to 20 mM glucose. The exposure times of different wells were stopped at ten time points (0, 1, 2, 3, 4, 5, 6, 8, 10 and 15 min) by lysing the cells with a butanol/methanol (3:1) solution according to the established BUME workflow. Subsequently, a 100 µL solution was removed and 10 µL of the solution was freeze-dried to remove the solvent and allow for reconstitution in any solvent. Here, the solvent systems that were selected for comparison were again the one-phase solvent system MeOH:H_2_O (9:1) and the BUME system. Reconstitution was achieved by adding the respective solvents to the freeze-dried material. Following, all samples (MeOH:H_2_O and the two BUME phases) were analyzed with DIP-MS or LLE DIP-MS, respectively, in both positive and negative modes. The analysis was performed using triplicates of all samples and in total 180 measurements were conducted, including blanks. Due to the high throughput with minimal required sample handling and analysis, all 180 samples were analyzed within 3 h. Following, data from all samples were extracted and analytes were putatively annotated based on accurate mass (<5 ppm) prior to plotting their relative intensities at the ten different time points to elucidate metabolome dynamics.

**Figure 4:**
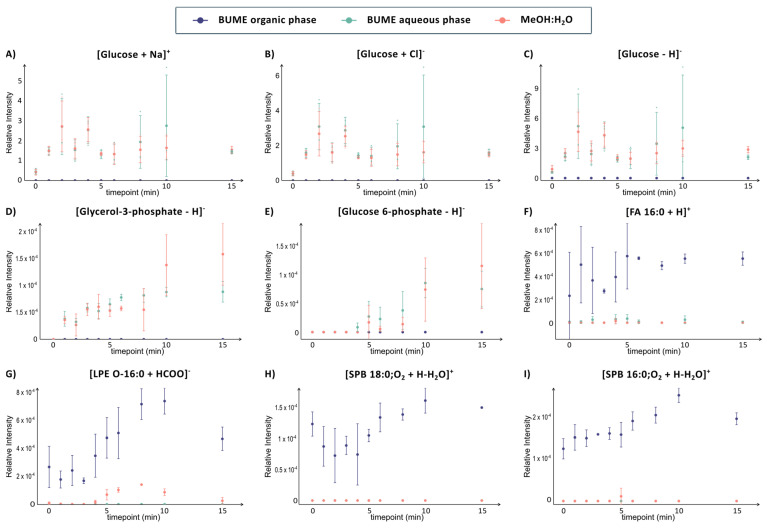
Time-resolved information on selected metabolites (**A**–**E**) and lipids (**F**–**I**) analyzed with the DIP-MS (orange) and the aqueous (green) and organic (purple) phases of the online LLE DIP-MS from INS-1 cells exposed to 20 mM glucose for different time durations (0, 1, 2, 3, 4, 5, 6, 8, 10 and 15 min). The glucose signal has been normalized to the signal of glucose-d_2_ while the rest have been normalized to the TIC. The error bars correspond to one standard deviation of triplicates of sample preparation.

The results show that time-resolved analysis provides extensive analyte and methodological information. For example, glucose was detected in three different mass channels across the two different ionization modes in both the aqueous-rich BUME phase and the MeOH:H_2_O solvent. Specifically, glucose was detected in negative ion mode both as deprotonated and chloride adduct and in positive ion mode as sodium adduct, in both the aqueous phase of BUME and in the MeOH:H_2_O solvent (Figure 4A–C). In all six cases, the time-resolved data show that the dynamics of glucose are equal, with a sharp increase within the first 2 min followed by more variation in the later time points (Figure 4A–C). The large error bars at the different time points could potentially be attributed to variations in cell number in the different samples or to differences in time of glucose exposure since this was performed manually. However, the consistent profile of intracellular glucose despite adduct ion and solvent composition suggests that the error bars originate from biological variability. Overall, the comprehensive analysis of several phases and adductions validates the dynamics of intracellular glucose in INS-1 cells.

The first intracellular metabolic pathway of glucose is glycolysis, where glucose is converted into pyruvate in several steps while generating energy in the form of ATP and NADPH. Glucose-6-phosphate and glycerol-3-phosphate are two intermediate metabolites in glycolysis that have been previously reported to be altered in glucose-stimulated INS-1 cells [62,63,64]. Both of these metabolites are detected as deprotonated ions in both the aqueous phase of BUME and the MeOH:H_2_O (9:1) using DIP-MS (Figure 4D,E). The time profiles of glucose-6-phosphate and glycerol-3-phosphate are very similar, with a constant increase of 1–4 min for each metabolite after glucose exposure. This suggests that glucose exposure increases the rate of glycolysis over time, similar to previous reports.

Of noteworthy, error bars of glucose-6-phosphate and glycerol-3-phosphate are overall smaller in the aqueous phase of the BUME compared to the MeOH:H_2_O solvent. This is reasonable due to their high solubility in water and their low detected intensity, which would make them more sensitive to ionization suppression by other analytes in the MeOH:H_2_O solvent. Even smaller error bars are found at the later time point for FA 16:0, 16:1 and 16:2, which all have similar profiles and are only detected in the organic-rich phase of BUME (Figure 4F and Figure S5). Finally, the low standard deviations at higher time points for the fatty acids indicate that biological variations in fatty acids after glucose exposure decrease after 5 min.

Although glucose, glucose-6-phosphate and glycerol-3-phosphate have been previously reported to be altered in glucose-stimulated INS-1 cells, this is the first report on minute resolved intracellular dynamics. LPE, which is also detected at low intensity in the MeOH:H_2_O solvent, is, to the best of our knowledge, the first report on its implication in insulin release (Figure 4G). Similarly, we report the dynamics of C16 and C18 sphinganines that are only detected in the organic phase. The intensity of both sphingosines increases between 5 and 10 min after exposure to glucose, which could be an indication of their importance in the transition into the second phase of insulin release (*p*-values after 6 min are between 0.001 and 0.033) (Figure 4H,I). Sphinganines can be linked to sphingolipid metabolism, which has been previously shown to be altered in patients with type 2 diabetes [65,66]. Although changes in sphingolipid metabolism have been reported in the glucose-exposed mouse insulinoma-6 (MIN6) cell line [67], our results are, to the best of our knowledge, the first to show these time-resolved alterations in sphingolipid metabolism in INS-1 cells. Overall, the results show that online LLE DIP-MS can be used to rapidly enable new insights into biological systems, such as the time-sensitive dynamics of the metabolome in INS-1 cells that are linked to insulin release.

## 4. Conclusions

Despite using non-traditional solvents for ESI-MS for the online LLE DIP-MS analysis, high coverage and detectability of various metabolite and lipid classes are found for all three LLE methods that were compared. Additionally, for all methods and phases, the DIP-MS is stable after only 10 s, enabling a rapid switch between phases. Out of the compared LLE methods, the BUME method was preferred since it showed high separation of lipids into the organic-rich phase and low solvent toxicity with more detected analytes than with a comparable one-phase system. The online LLE DIP-MS system can handle minute sample volumes since the phases do not need to be transferred after phase separation, which opens the opportunity to use samples with low cell counts. This is exemplified here by looking at time-resolved metabolomics and lipidomics from INS-1 cells exposed to high glucose with minute resolution. The separation into the two BUME phases prior to DIP-MS provides additional detectability of several lipids that show dynamic intracellular alterations in INS-1 cells exposed to glucose. Overall, we foresee the online LLE DIP-MS to be a useful tool when analyzing a large number of low-volume samples in a rapid manner and with high detectability of metabolites and lipids.

## Data Availability

Data will be available upon request.

## Supplementary Materials

The following supporting information can be downloaded at: https://www.mdpi.com/article/10.3390/metabo14110587/s1, Several tables, figures and notes detailing additional experimental conditions. Experimental section: Table S1: Concentrations (µM) of standard solutions used for the calibration curves in the organic phase of BUME liquid-liquid extraction. Table S2: Concentrations (µM) of standard solutions used for the calibration curves in the aqueous phase of BUME liquid-liquid extraction. Table S3: Mass spectrometric parameters used in the different experiments. Table S4: Analytes detected in positive mode from standard solution and in RBE analyzed with the different LLEs methods. Table S5: Analytes detected in negative mode from standard solution and in RBE analyzed with the different LLE methods. Table S6: Log *p* values of analytes and the corresponding extraction phase in the BUME extraction. Table S7: Analytes detected from INS-1 cell extraction in different LLE phases and in the one solvent method. Table S8: Analytes detected from INS-1 cell extraction in different LLE phases and in the one solvent method. Figure S1: Ionization efficiencies of analytes in solutions containing known concentrations of several molecules. Figure S2: Analytes detected in positive mode from INS-1 cells exposed to 20 mM Glucose for 15 min in the aqueous and organic LLE phases and in MeOH:H_2_O. Figure S3: Analytes detected in positive modes from INS-1 cells exposed to 20 mM Glucose for 15 min in the lower and upper LLE phases; and in MeOH:H_2_O solvent. Figure S4: Analytes detected in negative mode from INS-1 cells exposed to 20 mM Glucose for 15 min in the lower and upper LLE phases and in MeOH:H_2_O. Figure S5: Time resolved information of FA 16:1 (upper) and 16:2 (lower) analyzed with the DIP-MS (orange) and the aqueous (green) and organic (purple) phases of the online LLE DIP-MS from INS-1 cells exposed to 20 mM glucose for different times (0, 1, 2, 3, 4, 5, 6, 8, 10, 15 min).

## References

[1] Qiu S., Cai Y., Yao H., Lin C., Xie Y., Tang S., Zhang A. (2023). Small Molecule Metabolites: Discovery of Biomarkers and Therapeutic Targets. Signal Transduct. Target. Ther..

[2] Rinschen M.M., Ivanisevic J., Giera M., Siuzdak G. (2019). Identification of Bioactive Metabolites Using Activity Metabolomics. Nat. Rev. Mol. Cell Biol..

[3] Liu X., Locasale J.W. (2017). Metabolomics: A Primer. Trends Biochem. Sci..

[4] Yang W., Schoeman J.C., Di X., Lamont L., Harms A.C., Hankemeier T. (2024). A Comprehensive UHPLC-MS/MS Method for Metabolomics Profiling of Signaling Lipids: Markers of Oxidative Stress, Immunity and Inflammation. Anal. Chim. Acta.

[5] Pulliam A.N., Pybus A.F., Gaul D.A., Moore S.G., Wood L.B., Fern F.M., Laplaca M.C. (2024). Integrative Analysis of Cytokine and Lipidomics Datasets Following Mild Traumatic Brain Injury in Rats. Metabolites.

[6] Guo X.Y., Zhou J., Yu H., Cao H., Li X., Hu Q., Yu Y.Q. (2024). Serum Lipidomic Study of Long-Chain Fatty Acids in Psoriasis Patients Prior to and after Anti-IL-17A Monoclonal Antibody Treatment by Quantitative GC–MS Analysis with in Situ Extraction. Lipids Health Dis..

[7] Núñez-Sánchez M.Á., Martínez-Sánchez M.A., Martínez-Montoro J.I., Balaguer-Román A., Murcia-García E., Fernández-Ruiz V.E., Ferrer-Gómez M., Martínez-Cáceres C.M., Sledzinski T., Frutos M.D. (2024). Lipidomic Analysis Reveals Alterations in Hepatic FA Profile Associated With MASLD Stage in Patients With Obesity. J. Clin. Endocrinol. Metab..

[8] Shimada M., Miyagawa T., Kodama T., Toyoda H., Tokunaga K., Honda M. (2020). Metabolome Analysis Using Cerebrospinal Fluid from Narcolepsy Type 1 Patients. Sleep.

[9] Moldovan R.C., Bodoki E., Servais A.C., Chankvetadze B., Crommen J., Oprean R., Fillet M. (2018). Capillary Electrophoresis-Mass Spectrometry of Derivatized Amino Acids for Targeted Neurometabolomics—PH Mediated Reversal of Diastereomer Migration Order. J. Chromatogr. A.

[10] Dai Q., Xie P., Tan H., Zhang J., Wang F., Lei B., Cai Z. (2024). Metabolomics and Lipidomics with Mass Spectrometry Imaging Reveal Mechanistic Insights into Dibutyl Phthalate-Promoted Proliferation of Breast Cancer Cell Spheroids. Environ. Sci. Technol. Lett..

[11] Nizioł J., Ossoliński K., Płaza-Altamer A., Kołodziej A., Ossolińska A., Ossoliński T., Krupa Z., Ruman T. (2024). Untargeted Metabolomics of Bladder Tissue Using Liquid Chromatography and Quadrupole Time-of-Flight Mass Spectrometry for Cancer Biomarker Detection. J. Pharm. Biomed. Anal..

[12] Grebe S.K.G., Singh R.J. (2011). LC-MS/MS in the Clinical Laboratory—Where to from Here?. Clin. Biochem. Rev..

[13] CLSI (2022). Liquid Chromatography-Mass Spectrometry Methods.

[14] Fialkov A.B., Lehotay S.J., Amirav A. (2020). Less than One Minute Low-Pressure Gas Chromatography—Mass Spectrometry. J. Chromatogr. A.

[15] González-Domínguez R., García-Barrera T., Gómez-Ariza J.L. (2014). Using Direct Infusion Mass Spectrometry for Serum Metabolomics in Alzheimer’s Disease. Anal. Bioanal. Chem..

[16] Guo Y., Wang X., Qiu L., Qin X., Liu H., Wang Y., Li F., Wang X., Chen G., Song G. (2012). Probing Gender-Specific Lipid Metabolites and Diagnostic Biomarkers for Lung Cancer Using Fourier Transform Ion Cyclotron Resonance Mass Spectrometry. Clin. Chim. Acta.

[17] Heiles S. (2021). Advanced Tandem Mass Spectrometry in Metabolomics and Lipidomics—Methods and Applications. Anal. Bioanal. Chem..

[18] Draper J., Lloyd A.J., Goodacre R., Beckmann M. (2013). Flow Infusion Electrospray Ionisation Mass Spectrometry for High Throughput, Non-Targeted Metabolite Fingerprinting: A Review. Metabolomics.

[19] Floegel A., Kühn T., Sookthai D., Johnson T., Prehn C., Rolle-Kampczyk U., Otto W., Weikert C., Illig T., von Bergen M. (2018). Serum Metabolites and Risk of Myocardial Infarction and Ischemic Stroke: A Targeted Metabolomic Approach in Two German Prospective Cohorts. Eur. J. Epidemiol..

[20] Zukunft S., Sorgenfrei M., Prehn C., Möller G., Adamski J. (2013). Targeted Metabolomics of Dried Blood Spot Extracts. Chromatographia.

[21] Ekroos K., Chernushevich I.V., Simons K., Shevchenko A. (2002). Quantitative Profiling of Phospholipids by Multiple Precursor Ion Scanning on a Hybrid Quadrupole Time-of-Flight Mass Spectrometer. Anal. Chem..

[22] Schwudke D., Oegema J., Burton L., Entchev E., Hannich J.T., Ejsing C.S., Kurzchalia T., Shevchenko A. (2006). Lipid Profiling by Multiple Precursor and Neutral Loss Scanning Driven by the Data-Dependent Acquisition. Anal. Chem..

[23] Geromanos S., Philip J., Freckleton G., Tempst P. (1998). InJection Adaptable Fine Ionization Source (‘JaFIS’) for Continuous Flow Nano-Electrospray. Rapid Commun Mass Spectrom.

[24] Schwab N.V., Porcari A.M., Coelho M.B., Schmidt E.M., Jara J.L., Visentainer J.V., Eberlin M.N. (2012). Easy Dual-Mode Ambient Mass Spectrometry with Venturi Self-Pumping, Canned Air, Disposable Parts and Voltage-Free Sonic-Spray Ionization. Analyst.

[25] Santos V.G., Regiani T., Dias F.F.G., Romão W., Jara J.L.P., Klitzke C.F., Coelho F., Eberlin M.N. (2011). Venturi Easy Ambient Sonic-Spray Ionization. Anal. Chem..

[26] Tonin A.P.P., Poliseli C.B., Ribeiro M.A.S., Moraes L.A.B., Visentainer J.V., Eberlin M.N., Meurer E.C. (2018). Venturi Electrospray Ionization: Principles and Applications. Int. J. Mass Spectrom..

[27] Han J., Han F., Ouyang J., Li Q., Na N. (2013). Venturi-Electrosonic Spray Ionization Cataluminescence Sensor Array for Saccharides Detection. Anal. Chem..

[28] Marques C., Liu L., Duncan K.D., Lanekoff I. (2022). A Direct Infusion Probe for Rapid Metabolomics of Low-Volume Samples. Anal. Chem..

[29] Saini R.K., Prasad P., Shang X., Keum Y.S. (2021). Advances in Lipid Extraction Methods—A Review. Int. J. Mol. Sci..

[30] Folch J., Lees M., Stanley G.S. (1957). A Simple Method for the Isolation and Purification of Total Lipides from Animal Tissues. J. Biol. Chem..

[31] Bligh E.G., Dyer W.J. (1959). A rapid method of total lipid extraction and purification. Can. J. Biochem. Physiol..

[32] Bartolacci C., Andreani C., Vale G., Berto S., Melegari M., Crouch A.C., Baluya D.L., Kemble G., Hodges K., Starrett J. (2022). Targeting de Novo Lipogenesis and the Lands Cycle Induces Ferroptosis in KRAS-Mutant Lung Cancer. Nat. Commun..

[33] Li Y., Bansal S., Sridharan V., Bansal S., Jayatilake M.M., Fernández J.A., Griffin J.H., Boerma M., Cheema A.K. (2023). Urinary Metabolomics for the Prediction of Radiation-Induced Cardiac Dysfunction. Metabolites.

[34] Löfgren L., Ståhlman M., Forsberg G.-B., Saarinen S., Nilsson R., Hansson G.I. (2012). The BUME Method: A Novel Automated Chloroform-Free 96-Well Total Lipid Extraction Method for Blood Plasma. J. Lipid Res..

[35] Rosqvist F., Kullberg J., Ståhlman M., Cedernaes J., Heurling K., Johansson H.E., Iggman D., Wilking H., Larsson A., Eriksson O. (2019). Overeating Saturated Fat Promotes Fatty Liver and Ceramides Compared with Polyunsaturated Fat: A Randomized Trial. J. Clin. Endocrinol. Metab..

[36] Vale G., Martin S.A., Mitsche M.A., Thompson B.M., Eckert K.M., McDonald J.G. (2019). Three-Phase Liquid Extraction: A Simple and Fast Method for Lipidomic Workflows. J. Lipid Res..

[37] Shibusawa Y., Yamakawa Y., Noji R., Yanagida A., Shindo H., Ito Y. (2006). Three-Phase Solvent Systems for Comprehensive Separation of a Wide Variety of Compounds by High-Speed Counter-Current Chromatography. J. Chromatogr. A.

[38] Sinturel F., Chera S., Brulhart-Meynet M.C., Montoya J.P., Stenvers D.J., Bisschop P.H., Kalsbeek A., Guessous I., Jornayvaz F.R., Philippe J. (2023). Circadian Organization of Lipid Landscape Is Perturbed in Type 2 Diabetic Patients. Cell Reports Med..

[39] Sündermann A., Eggers L.F., Schwudke D. (2016). Liquid Extraction: Bligh and Dyer. Encyclopedia of Lipidomics.

[40] Pluskal T., Castillo S., Villar-Briones A., Orešič M. (2010). MZmine 2: Modular Framework for Processing, Visualizing, and Analyzing Mass Spectrometry-Based Molecular Profile Data. BMC Bioinform..

[41] RStudio Team (2020). RStudio: Integrated Development for R.

[42] Kebarle P., Tang L. (1993). From Ions in Solution To Ions in the Gas Phase. Anal. Chem..

[43] Little J.L., Wempe M.F., Buchanan C.M. (2006). Liquid Chromatography-Mass Spectrometry/Mass Spectrometry Method Development for Drug Metabolism Studies: Examining Lipid Matrix Ionization Effects in Plasma. J. Chromatogr. B Anal. Technol. Biomed. Life Sci..

[44] Jemal M., Ouyang Z., Xia Y.Q. (2010). Systematic LC-MS/MS Bioanalytical Method Development That Incorporates Plasma Phospholipids Risk Avoidance, Usage of Incurred Sample and Well Thought-out Chromatography. Biomed. Chromatogr..

[45] Guo X., Lankmayr E. (2011). Phospholipid-Based Matrix Effects in LC-MS Bioanalysis. Bioanalysis.

[46] Smedes F., Askland T.K. (1999). Revisiting the Development of the Bligh and Dyer Total Lipid Determination Method. Mar. Pollut. Bull..

[47] Leo A., Hansch C., Elkins D. (1971). Partition Coefficients and Their Uses. Chem. Rev..

[48] Cech N.B., Enke C.G. (2001). Practical Implications of Some Recent Studies in Electrospray Ionization Fundamentals. Mass Spectrom. Rev..

[49] Konermann L., Ahadi E., Rodriguez A.D., Vahidi S. (2013). Unraveling the Mechanism of Electrospray Ionization. Anal. Chem..

[50] Kebarle P., Verkerk U.H. (2009). Electrospray: From Ions in Solution to Ions in the Gas Phase, What We Know Now. Mass Spectrom. Rev..

[51] Tang L., Kebarle P. (1993). Dependence of Ion Intensity in Electrospray Mass Spectrometry on the Concentration of the Analytes in the Electrosprayed Solution. Anal. Chem..

[52] Zhou S., Cook K.D. (2001). A Mechanistic Study of Electrospray Mass Spectrometry: Charge Gradients within Electrospray Droplets and Their Influence on Ion Response. J. Am. Soc. Mass Spectrom..

[53] Turtoi E., Jeudy J., Henry S., Dadi I., Valette G., Enjalbal C., Turtoi A. (2023). Analysis of Polar Primary Metabolites in Biological Samples Using Targeted Metabolomics and LC-MS. STAR Protoc..

[54] Sharaf B.M., Giddey A.D., Alniss H., Al-Hroub H.M., El-Awady R., Mousa M., Almehdi A., Soares N.C., Semreen M.H. (2022). Untargeted Metabolomics of Breast Cancer Cells MCF-7 and SkBr3 Treated With Tamoxifen/Trastuzumab. Cancer Genom. Proteom..

[55] Southam A.D., Weber R.J.M., Engel J., Jones M.R., Viant M.R. (2017). A Complete Workflow for High-Resolution Spectral-Stitching Nanoelectrospray Direct-Infusion Mass-Spectrometry-Based Metabolomics and Lipidomics. Nat. Protoc..

[56] Kirwan J.A., Weber R.J.M., Broadhurst D.I., Viant M.R. (2014). Direct Infusion Mass Spectrometry Metabolomics Dataset: A Benchmark for Data Processing and Quality Control. Sci. Data.

[57] Zahn J.A., Higgs R.E., Hilton M.D. (2001). Use of Direct-Infusion Electrospray Mass Spectrometry to Guide Empirical Development of Improved Conditions for Expression of Secondary Metabolites from Actinomycetes. Appl. Environ. Microbiol..

[58] Lin L., Yu Q., Yan X., Hang W., Zheng J., Xing J., Huang B. (2010). Direct Infusion Mass Spectrometry or Liquid Chromatography Mass Spectrometry for Human Metabonomics? A Serum Metabonomic Study of Kidney Cancer. Analyst.

[59] Pöhö P., Lipponen K., Bespalov M.M., Sikanen T., Kotiaho T., Kostiainen R. (2019). Comparison of Liquid Chromatography-Mass Spectrometry and Direct Infusion Microchip Electrospray Ionization Mass Spectrometry in Global Metabolomics of Cell Samples. Eur. J. Pharm. Sci..

[60] Spégel P., Mulder H. (2020). Metabolomics Analysis of Nutrient Metabolism in β-Cells. J. Mol. Biol..

[61] Gerich J.E. (2002). Is Reduced First-Phase Insulin Release the Earliest Detectable Abnormality in Individuals Destined to Develop Type 2 Diabetes?. Diabetes.

[62] Spégel P., Sharoyko V.V., Goehring I., Danielsson A.P.H., Malmgren S., Nagorny C.L.F., Andersson L.E., Koeck T., Sharp G.W.G., Straub S.G. (2013). Time-Resolved Metabolomics Analysis of β-Cells Implicates the Pentose Phosphate Pathway in the Control of Insulin Release. Biochem. J..

[63] Andersson L.E., Shcherbina L., Al-Majdoub M., Vishnu N., Arroyo C.B., Carrara J.A., Wollheim C.B., Fex M., Mulder H., Wierup N. (2018). Glutamine-Elicited Secretion of Glucagon-like Peptide 1 Is Governed by an Activated Glutamate Dehydrogenase. Diabetes.

[64] Lorenz M.A., El Azzouny M.A., Kennedy R.T., Burant C.F. (2013). Metabolome Response to Glucose in the β-Cell Line INS-1832/13. J. Biol. Chem..

[65] Adams J.M., Pratipanawatr T., Berria R., Wang E., DeFronzo R.A., Sullards M.C., Mandarino L.J. (2004). Ceramide Content Is Increased in Skeletal Muscle from Obese Insulin-Resistant Humans. Diabetes.

[66] Summers S.A. (2010). Sphingolipids and Insulin Resistance: The Five Ws. Curr. Opin. Lipidol..

[67] Stanford J.C., Morris A.J., Sunkara M., Popa G.J., Larson K.L., Özcan S. (2012). Sphingosine 1-Phosphate (S1P) Regulates Glucose-Stimulated Insulin Secretion in Pancreatic Beta Cells. J. Biol. Chem..

