# Supplementary material for: Online Direct Infusion Mass Spectrometry of Liquid–Liquid Extraction Phases for Metabolite and Lipid Profiling with the Direct Infusion Probe
Source: Metabolites. 2024 Oct 30;14(11):587. doi: 10.3390/metabo14110587 (PMC11596504; doi:10.3390/metabo14110587)
Supplement: Supplementary file 1 [file metabolites-14-00587-s001.zip › metabolites-3256656-supplementary.pdf]

Supporting Information

Online direct infusion mass spectrometry of LLE phases for metabolite and lipid profiling with the direct infusion probe

Cátia Marques[1], Lena Blaase[1], Ingela Lanekoff[1]*

[1] Department of Chemistry – BMC, Uppsala University, 75123 Uppsala, Sweden

Corresponding author:
Prof. Ingela Lanekoff
Ingela.Lanekoff@kemi.uu.se
Dept. of Chemistry-BMC (576)
Uppsala University
751 23 Uppsala
Sweden

Experimental section

Cell counting: Cells from 4 different wells were washed with water once and detached by adding trypsin for 3 min. Culture medium was added and the cells were counted using staining with trypan blue and a Bürker chamber. The cells were counter in 8 non-touching squares. Across the 4 wells, 405750 cells were counted with 6% RSD.

Data extraction with MZmine 2.53: The raw files were imported to MZmine 2.53. A mass list of all m/z detected across all files was performed using all scans in the files and a considering a noise level of 1E3 and 1E4 for data acquired in negative and positive modes, respectively. Chromatograms were built over the whole time of each file with a minimum time span of 0.1 min, minimum height of 1E3 and 1E4 for data acquired in negative and positive modes, respectively. Moreover, an m/z tolerance of 0.001 (or 5 ppm) was considered for this purpose. The chromatograms were deisotoped considering the mass tolerance previously mentioned, retention time tolerance or 1 min and maximum charge of 1. The isotope with highest intensity was kept. The deisotoped chromatograms were used to perform peak alignment using the Join alignment method where an m/z tolerance of 0.001 (or 5ppm) was applied, the weight for m/z was 100, the retention time tolerance was 1 min and the weight for retention time was 0. The peak identification was performed with a Custom database search using a file containing identification and theoretical m/z for different analytes and respective adducts, considering the m/z tolerance mentioned above and retention time tolerance of 1 min. The results were exported and further analyzed in excel.

Parameters for inclusion of annotated analytes for bubble plot: For an analyte to be considered, the average of the samples needed to be 1.3-times higher than the average of the blank for each solvent, and the signal analyte would have to be detected in all triplicates analyzed for the LLE conditions and in all. Only one sample of RBE was prepared in MeOH:H_2O, thus this sample that to be 1.3-times higher than the blank. Additionally, the blank solutions had known solutions of amino acids, D9-FA 18:1, LPC 19:0, PC 11:0/11:0, FA 16:1, FA 18:1, FA 20:1, FA 20:4, FA 20:5, and PC 36:1. Thus, if these analytes were detected in the blanks and the RBE samples with similar signal, the peaks were considered for the bubble plot.

Parameters for inclusion of annotated analytes for analytes detected in INS-1 prepared with BUME LLE and methanolic solution: For an analyte to be considered, the average of the samples needed to be 1.3-times higher than the average of the blank for each solvent.

Table S1. Concentrations (μM) of standard solutions used for the calibration curves in the organic phase of BUME liquid-liquid extraction.

	Sol 1	Sol 2	Sol 3	Sol 4	Sol 5	Sol 6	Sol 7	Sol 8	Sol 9	Sol 10
**Ala**	0.1058	0.2782	0.8328	1.3759	2.1962	3.2455	4.3854	5.5310	8.3056	11.0377
**Arg**	0.1028	0.2702	0.8086	1.3360	2.1325	3.1513	4.2581	5.3705	8.0645	10.7174
**Asn**	0.0786	0.2066	0.6186	1.0219	1.6312	2.4106	3.2572	4.1081	6.1689	8.1982
**Asp**	0.1816	0.4772	1.4285	2.3601	3.7672	5.5671	7.5223	9.4874	14.2466	18.9331
**Cys**	0.1934	0.5083	1.5216	2.5138	4.0126	5.9297	8.0123	10.1053	15.1746	20.1664
**Glu**	0.1925	0.5059	1.5144	2.5019	3.9936	5.9016	7.9744	10.0575	15.1028	20.0710
**Gln**	0.0834	0.2192	0.6562	1.0841	1.7305	2.5572	3.4554	4.3580	6.5442	8.6970
**Gly**	0.1610	0.4231	1.2665	2.0924	3.3399	4.9355	6.6690	8.4111	12.6305	16.7854
**His**	0.1400	0.3680	1.1015	1.8197	2.9047	4.2925	5.8001	7.3152	10.9849	14.5984
**Ile**	0.0123	0.0323	0.0967	0.1597	0.2549	0.3768	0.5091	0.6421	0.9641	1.2813
**Leu**	0.0123	0.0322	0.0965	0.1594	0.2544	0.3760	0.5080	0.6407	0.9622	1.2787
**Lys**	0.0683	0.1795	0.5372	0.8874	1.4165	2.0933	2.8285	3.5674	5.3570	7.1192
**Met**	0.2752	0.7233	2.1652	3.5771	5.7099	8.4378	11.4014	14.3797	21.5932	28.6965
**Phe**	0.0109	0.0288	0.0861	0.1422	0.2270	0.3354	0.4532	0.5716	0.8584	1.1408
**Pro**	0.0063	0.0167	0.0499	0.0825	0.1317	0.1946	0.2629	0.3316	0.4980	0.6618
**Ser**	0.1807	0.4751	1.4221	2.3495	3.7503	5.5421	7.4886	9.4447	14.1826	18.8481
**Thr**	0.0593	0.1559	0.4667	0.7710	1.2307	1.8187	2.4574	3.0994	4.6541	6.1852
**Trp**	0.0052	0.0137	0.0410	0.0678	0.1082	0.1598	0.2160	0.2724	0.4090	0.5436
**Tyr**	0.0098	0.0258	0.0772	0.1276	0.2036	0.3009	0.4066	0.5128	0.7701	1.0234
**Val**	0.3730	0.9803	2.9344	4.8479	7.7383	11.4354	15.4518	19.4882	29.2643	38.8909
**GABA**	0.1194	0.3139	0.9397	1.5525	2.4781	3.6621	4.9483	6.2409	9.3716	12.4545
**Creatine**	0.1780	0.4678	1.4002	2.3133	3.6925	5.4567	7.3732	9.2992	13.9641	18.5577
**Choline**	0.0011	0.0028	0.0084	0.0139	0.0222	0.0329	0.0444	0.0560	0.0841	0.1117
**Carnitine**	0.0005	0.0013	0.0039	0.0064	0.0103	0.0152	0.0205	0.0259	0.0389	0.0517
**Acetylcholine**	0.0003	0.0007	0.0022	0.0036	0.0058	0.0086	0.0116	0.0146	0.0219	0.0292
**FA 18:1**	0.0114	0.0299	0.0896	0.1480	0.2362	0.3815	0.5155	0.6502	0.9763	1.2975
**FA 20:1**	0.0070	0.0184	0.0550	0.0908	0.1450	0.2121	0.2866	0.3614	0.5428	0.7213
**PC 36:1**	0.0280	0.0737	0.2207	0.3646	0.5819	1.0655	1.4397	1.8158	2.7267	3.6237
**Ala-15N**	1.7393	1.7607	1.7632	1.7383	1.7473	1.7374	1.7468	1.7713	1.7473	1.7603
**Arg-15N**	0.3479	0.3521	0.3526	0.3477	0.3495	0.3475	0.3494	0.3543	0.3495	0.3521
**Asn-15N**	1.9481	1.9720	1.9748	1.9469	1.9570	1.9459	1.9564	1.9838	1.9570	1.9715
**Asp-15N**	1.9481	1.9720	1.9748	1.9469	1.9570	1.9459	1.9564	1.9838	1.9570	1.9715
**Cys-15N**	0.4638	0.4695	0.4702	0.4636	0.4659	0.4633	0.4658	0.4723	0.4660	0.4694
**Glu-15N**	1.1132	1.1268	1.1285	1.1125	1.1183	1.1119	1.1179	1.1336	1.1183	1.1266
**Gln-15N**	0.4638	0.4695	0.4702	0.4636	0.4659	0.4633	0.4658	0.4723	0.4660	0.4694
**Gly-15N**	1.3451	1.3616	1.3635	1.3443	1.3513	1.3436	1.3508	1.3698	1.3513	1.3613
**His-15N**	0.1160	0.1174	0.1175	0.1159	0.1165	0.1158	0.1165	0.1181	0.1165	0.1174
**Ile-15N**	0.6494	0.6573	0.6583	0.6490	0.6523	0.6486	0.6521	0.6613	0.6523	0.6572
**Leu-15N**	1.0436	1.0564	1.0579	1.0430	1.0484	1.0424	1.0481	1.0628	1.0484	1.0562
**Lys-15N**	0.3942	0.3991	0.3997	0.3940	0.3961	0.3938	0.3959	0.4015	0.3961	0.3990
**Met-15N**	0.1855	0.1878	0.1881	0.1854	0.1864	0.1853	0.1863	0.1889	0.1864	0.1878
**Phe-15N**	0.4638	0.4695	0.4702	0.4636	0.4659	0.4633	0.4658	0.4723	0.4660	0.4694
**Pro-15N**	0.4638	0.4695	0.4702	0.4636	0.4659	0.4633	0.4658	0.4723	0.4660	0.4694
**Ser-15N**	0.6725	0.6808	0.6818	0.6722	0.6756	0.6718	0.6754	0.6849	0.6756	0.6806
**Thr-15N**	0.7653	0.7747	0.7758	0.7649	0.7688	0.7645	0.7686	0.7794	0.7688	0.7745
**Trp-15N**	0.4638	0.4695	0.4702	0.4636	0.4659	0.4633	0.4658	0.4723	0.4660	0.4694
**Tyr-15N**	0.2319	0.2348	0.2351	0.2318	0.2330	0.2317	0.2329	0.2362	0.2330	0.2347
**Val-15N**	0.8349	0.8451	0.8463	0.8344	0.8387	0.8340	0.8384	0.8502	0.8387	0.8449
**FA18:1-d_9**	0.1906	0.1929	0.1932	0.1905	0.1915	0.1904	0.1914	0.1941	0.1915	0.1929
**LPC 19:0**	0.3216	0.3256	0.3261	0.3215	0.3231	0.3213	0.3230	0.3275	0.3231	0.3255
**PC11:0/11:0**	0.4991	0.5052	0.5059	0.4988	0.5014	0.4985	0.5012	0.5082	0.5014	0.5051
**PE 30:0**	2.7364	2.7700	2.7740	2.7348	2.7489	2.7334	2.7481	2.7866	2.7490	2.7694
**PG 30:0**	3.4453	3.4876	3.4926	3.4433	3.4611	3.4415	3.4601	3.5086	3.4612	3.4868
**Glutamate-d_3**	1.1618	1.1761	1.1778	1.1611	1.1671	1.1605	1.1668	1.1831	1.1672	1.1758
**Acetylcholine-d_9**	0.9470	0.9587	0.9600	0.9465	0.9514	0.9460	0.9511	0.9644	0.9514	0.9584
**GABA-d_2**	0.6854	0.6938	0.6948	0.6850	0.6885	0.6846	0.6883	0.6979	0.6885	0.6936
**Carnitine-d_3**	0.0468	0.0474	0.0475	0.0468	0.0471	0.0468	0.0470	0.0477	0.0471	0.0474

Table S2. Concentrations (μM) of standard solutions used for the calibration curves in the aqueous phase of BUME liquid-liquid extraction.

	Sol 1	Sol 2	Sol 3	Sol 4	Sol 5	Sol 6	Sol 7	Sol 8	Sol 9	Sol 10
**Ala**	0.1072	0.2829	0.8204	1.3374	2.2240	3.3588	4.4656	5.5659	8.2723	10.8161
**Arg**	0.1041	0.2747	0.7966	1.2986	2.1595	3.2614	4.3360	5.4044	8.0322	10.5022
**Asn**	0.0796	0.2101	0.6094	0.9933	1.6519	2.4948	3.3168	4.1341	6.1442	8.0336
**Asp**	0.1839	0.4852	1.4073	2.2940	3.8149	5.7615	7.6599	9.5473	14.1896	18.5529
**Cys**	0.1958	0.5168	1.4990	2.4435	4.0634	6.1367	8.1588	10.1692	15.1138	19.7614
**Glu**	0.1949	0.5144	1.4919	2.4319	4.0442	6.1077	8.1202	10.1211	15.0423	19.6679
**Gln**	0.0845	0.2229	0.6464	1.0538	1.7524	2.6465	3.5186	4.3856	6.5180	8.5223
**Gly**	0.1630	0.4302	1.2477	2.0338	3.3822	5.1079	6.7909	8.4642	12.5799	16.4483
**His**	0.1418	0.3741	1.0851	1.7688	2.9415	4.4424	5.9061	7.3615	10.9409	14.3053
**Ile**	0.0124	0.0328	0.0952	0.1552	0.2582	0.3899	0.5184	0.6461	0.9603	1.2556
**Leu**	0.0124	0.0328	0.0950	0.1549	0.2576	0.3891	0.5173	0.6448	0.9583	1.2530
**Lys**	0.0691	0.1825	0.5292	0.8626	1.4345	2.1664	2.8802	3.5900	5.3355	6.9762
**Met**	0.2787	0.7354	2.1330	3.4770	5.7822	8.7325	11.6098	14.4706	21.5068	28.1202
**Phe**	0.0111	0.0292	0.0848	0.1382	0.2299	0.3471	0.4615	0.5752	0.8549	1.1178
**Pro**	0.0064	0.0170	0.0492	0.0802	0.1333	0.2014	0.2677	0.3337	0.4960	0.6485
**Ser**	0.1830	0.4830	1.4010	2.2837	3.7978	5.7356	7.6254	9.5044	14.1258	18.4696
**Thr**	0.0601	0.1585	0.4597	0.7494	1.2463	1.8822	2.5024	3.1189	4.6355	6.0610
**Trp**	0.0053	0.0139	0.0404	0.0659	0.1095	0.1654	0.2199	0.2741	0.4074	0.5326
**Tyr**	0.0099	0.0262	0.0761	0.1240	0.2062	0.3114	0.4141	0.5161	0.7670	1.0029
**Val**	0.3777	0.9967	2.8908	4.7122	7.8363	11.8347	15.7343	19.6113	29.1471	38.1100
**GABA**	0.1210	0.3192	0.9257	1.5090	2.5095	3.7900	5.0388	6.2803	9.3341	12.2044
**Creatine**	0.1802	0.4756	1.3794	2.2485	3.7393	5.6472	7.5080	9.3580	13.9082	18.1850
**Choline**	0.0011	0.0029	0.0083	0.0135	0.0225	0.0340	0.0452	0.0564	0.0838	0.1095
**Carnitine**	0.0005	0.0013	0.0038	0.0063	0.0104	0.0157	0.0209	0.0261	0.0387	0.0506
**Acetylcholine**	0.0003	0.0007	0.0022	0.0035	0.0059	0.0089	0.0118	0.0147	0.0219	0.0286
**FA 18:1**	0.0107	0.0273	0.0727	0.1099	0.1620	0.3509	0.4583	0.5616	0.8002	1.0080
**FA 20:1**	0.0065	0.0167	0.0446	0.0675	0.0995	0.1951	0.2548	0.3122	0.4448	0.5604
**PC 36:1**	0.0263	0.0672	0.1790	0.2707	0.3993	0.9799	1.2801	1.5686	2.2348	2.8152
**Ala-15N**	1.7375	1.7777	1.7427	1.6958	1.7690	1.7806	1.7609	1.7508	1.7590	1.7351
**Arg-15N**	0.3475	0.3555	0.3485	0.3392	0.3538	0.3561	0.3522	0.3502	0.3518	0.3470
**Asn-15N**	1.9461	1.9910	1.9519	1.8993	1.9813	1.9942	1.9722	1.9609	1.9701	1.9433
**Asp-15N**	1.9461	1.9910	1.9519	1.8993	1.9813	1.9942	1.9722	1.9609	1.9701	1.9433
**Cys-15N**	0.4633	0.4741	0.4647	0.4522	0.4717	0.4748	0.4696	0.4669	0.4691	0.4627
**Glu-15N**	1.1120	1.1377	1.1154	1.0853	1.1322	1.1396	1.1270	1.1205	1.1258	1.1104
**Gln-15N**	0.4633	0.4741	0.4647	0.4522	0.4717	0.4748	0.4696	0.4669	0.4691	0.4627
**Gly-15N**	1.3437	1.3748	1.3477	1.3114	1.3680	1.3770	1.3618	1.3539	1.3603	1.3418
**His-15N**	0.1158	0.1185	0.1162	0.1131	0.1179	0.1187	0.1174	0.1167	0.1173	0.1157
**Ile-15N**	0.6487	0.6637	0.6506	0.6331	0.6604	0.6647	0.6574	0.6536	0.6567	0.6478
**Leu-15N**	1.0425	1.0666	1.0456	1.0175	1.0614	1.0683	1.0566	1.0505	1.0554	1.0410
**Lys-15N**	0.3938	0.4029	0.3950	0.3844	0.4010	0.4036	0.3991	0.3968	0.3987	0.3933
**Met-15N**	0.1853	0.1896	0.1859	0.1809	0.1887	0.1899	0.1878	0.1868	0.1876	0.1851
**Phe-15N**	0.4633	0.4741	0.4647	0.4522	0.4717	0.4748	0.4696	0.4669	0.4691	0.4627
**Pro-15N**	0.4633	0.4741	0.4647	0.4522	0.4717	0.4748	0.4696	0.4669	0.4691	0.4627
**Ser-15N**	0.6719	0.6874	0.6739	0.6557	0.6840	0.6885	0.6809	0.6770	0.6802	0.6709
**Thr-15N**	0.7645	0.7822	0.7668	0.7461	0.7784	0.7834	0.7748	0.7703	0.7740	0.7634
**Trp-15N**	0.4633	0.4741	0.4647	0.4522	0.4717	0.4748	0.4696	0.4669	0.4691	0.4627
**Tyr-15N**	0.2317	0.2370	0.2324	0.2261	0.2359	0.2374	0.2348	0.2334	0.2345	0.2313
**Val-15N**	0.8340	0.8533	0.8365	0.8140	0.8491	0.8547	0.8452	0.8404	0.8443	0.8328
**FA18:1-d$_9$**	0.1904	0.1948	0.1910	0.1858	0.1939	0.1951	0.1930	0.1919	0.1928	0.1901
**LPC 19:0**	0.3213	0.3287	0.3223	0.3136	0.3271	0.3293	0.3256	0.3238	0.3253	0.3208
**PC11:0/11:0**	0.4986	0.5101	0.5000	0.4866	0.5076	0.5109	0.5053	0.5024	0.5047	0.4978
**PE 30:0**	2.7336	2.7968	2.7417	2.6679	2.7831	2.8013	2.7704	2.7544	2.7674	2.7297
**PG 30:0**	3.4418	3.5214	3.4521	3.3591	3.5041	3.5270	3.4881	3.4680	3.4843	3.4369
**Glutamate-d$_3$**	1.1606	1.1874	1.1641	1.1327	1.1816	1.1894	1.1762	1.1695	1.1750	1.1590
**Acetylcholine-d$_9$**	0.9461	0.9679	0.9489	0.9233	0.9632	0.9695	0.9588	0.9533	0.9578	0.9447
**GABA-d$_2$**	0.6847	0.7005	0.6867	0.6682	0.6971	0.7016	0.6939	0.6899	0.6931	0.6837
**Carnitine-d$_3$**	0.0468	0.0479	0.0469	0.0457	0.0476	0.0480	0.0474	0.0472	0.0474	0.0467

Table S3: Mass spectrometric parameters used in the different experiments.

Voltage applied					
**Ionization mode**	LLE Phase	Bligh-Dyer (kV)	BUME (kV)	3-PLE (kV)	MeOH/ H_2O (kV)
**Positive mode**	Upper phase	2.8	2.3	2.4	2.6
	Middle phase	-	-	2.6	
	Lower phase	2.7	2.2	2.8	
**Negative mode**	Upper phase	2.5	2.0	2.1	2.3
	Middle phase	-	-	2.4	
	Lower phase	2.4	1.9	2.5	

**Mass range for BUME experiments**		
**Positive mode**	Characterization	*m/z* 75-1000
	INS-1 application	*m/z* 75-400
**Negative mode**	Characterization	*m/z* 75-1000
	INS-1 application	*m/z* 100-1000
**Mass range for MeOH:H_2O experiments**		
**Positive and negative modes**		*m/z* 75-1000

Table S4. Analytes detected in positive mode from standard solution and in RBE analyzed with the different LLEs methods. The analytes were putatively annotated based on mass accuracy (< 5 ppm) and are divided in subcategories.

	BD up	BD low	BUME up	BUME low	PLE up	PLE mid	PLE low
**Essential amino acids**							
[15N-Ala + H]+	1			1			1
[15N-Asn + H]+	1		1	1			1
[15N-Cys + H]+	1			1			1
[15N-Gln + H]+	1		1	1			1
[15N-Glu + Na]+	1						1
[15N-His + H]+	1			1			1
[15N-Iso + H]+	1		1	1			1
[15N-Lys + H]+	1			1			1
[15N-Met + H]+	1			1			1
[15N-Phe + Na]+							

[15N-Pro + H]+	1		1	1			1
[15N-Thr + Na]+	1						
[15N-Try + H]+	1		1	1			1
[15N-Val + H]+	1		1	1			1
[Ala + H]+	1						1
[Asp + H]+			1				
[Gln + H]+			1				
[Glu + Na]+							1
[Gly + H]+							1
[Pro + H]+							1
Sum	*14*	*0*	*8*	*11*	*0*	*0*	*16*
**Other small metabolites**							
[Acetylcholine]+							1
[Choline]+	1			1			1
[Creatine + H]+	1		1	1			1
[Creatinine + H]+	1						1
[Cystathionine + H]+				1			
[GABA + H]+	1			1			1
[Glucosamine + H-H_2O]+							1
[Glucose + Na]+	1			1			1
[Glycerate + Na]+							1
[Glycerol + Na]+							1
[Hydroxyproline + H]+							1
[Hypoxanthine + H]+	1		1	1			1
[Inosine + Na]+	1			1			1
[Lactate + Na]+	1						
[N-acetylaspartate + Na]+	1		1	1			1
[Nicotinamide + H]+	1		1				1
[Phosphocholine]+	1	1	1				
[Taurine + H]+							1
[Threonate + Na]+							1
[Xanthine + H]+			1				
Sum	*11*	*1*	*6*	*8*	*0*	*0*	*16*
**Ceramides**							
[Cer 34:0;O + Na]+			1				
[Cer 36:1;O2 + H]+			1				
Sum	*0*	*0*	*2*	*0*	*0*	*0*	*0*
**DG**							
[DG 34:1 + H-H_2O]+			1				
[DG 36:1 + H-H_2O]+		1	1				
[DG 36:2 + H-H_2O]+			1				

[DG 38:4 + H-H_2O]+		1	1				
[DG 38:5 + H-H_2O]+			1				
[DG 38:6 + H-H_2O]+			1				
[DG 40:4 + H-H_2O]+			1				
[DG 40:5 + H-H_2O]+			1				
[DG 40:6 + H-H_2O]+		1	1				
[DG;O-34:1 + H-H_2O]+			1				
Sum	*0*	*3*	*10*	*0*	*0*	*0*	*0*
**Fatty acyls**							
[FA 4:0;O_2 + Na]+	1						
[FA 4:1 + H]+	1			1			
[FA 6:0 + H]+				1		1	
[FA 6:0;O + Na]+				1			1
[FA 6:0;O_2 + Na]+							1
[FA 6:1 + H]+				1		1	
[FA 6:1;O + H]+							1
[FA 6:1;O_2 + Na]+							
[FA 8:0 + H]+				1			
[FA 8:0;O + H]+		1					
[FA 8:1 + H]+				1			
[FA 8:1;O + Na]+							1
[FA 8:1;O_2 + Na]+				1			
[FA 8:2 + H]+				1			
[FA 8:2 + Na]+	1						
[FA 8:2;O + Na]+				1			
[FA 8:2;O_2 + Na]+							
[FA 10:0;O + Na]+							
[FA 10:1 + Na]+				1			
[FA 10:2;O + H]+		1					
[FA 10:2;O_2 + H]+							
[FA 10:4 + H]+				1			
[FA 10:5 + H]+				1		1	
[FA 10:6;O + H]+	1					1	
[FA 12:1 + H]+				1			
[FA 12:1;O_3 + Na]+							
[FA 12:2 + H]+							
[FA 12:2;O_2 + H]+						1	
[FA 12:2;O_2 + Na]+							
[FA 12:3 + H]+		1					
[FA 12:3;O + H]+		1					
[FA 12:4 + H]+						1	
[FA 12:5;O_2 + H]+							
[FA 12:6;O + H]+				1		1	

[FA 14:0 + H]+				1			
[FA 14:1 + H]+							
[FA 14:1;O_2 + H]+		1					
[FA 14:4 + H]+				1			
[FA 16:0 + H]+				1		1	
[FA 16:1 + H]+							
[FA 16:3 + H]+						1	
[FA 16:4 + H]+		1				1	
[FA 16:4;O + H]+						1	
[FA 18:2 + H]+		1					
[FA 18:3 + H]+		1				1	
[FA 22:1;O_2 + H]+						1	
[FAL 6:0 + H]+						1	
[FAL 6:2 + H]+				1			
[FAL 8:2 + H]+				1		1	
[FAL 8:3 + H]+				1			
[FAL 10:2 + H]+				1		1	
[FAL 10:3 + H]+				1		1	
[FAL 12:1 + H]+							
[FAL 12:2 + H]+						1	
[FAL 12:5 + H]+						1	
[FAL 14:3 + H]+						1	
[FAL 16:3 + H]+				1		1	
[FAL 18:2 + H]+		1					
[FAL 18:3 + H]+		1					
Sum	*1*	*9*	*0*	*14*	*0*	*19*	*0*
**LPC**							
[LPC 16:0 + H]+		1	1			1	
[LPC 18:0 + H]+		1	1			1	
[LPC 18:1 + H]+		1	1			1	
[LPC 19:0 + H]+		1	1			1	
[LPC 20:1 + H]+			1				
[LPC 20:4 + H]+		1	1				
[LPC 22:6 + H]+		1	1				
Sum	*0*	*6*	*7*	*0*	*0*	*4*	*0*
**LPE**							
[LPE 14:0 + H]+			1				
[LPE 16:0 + H]+		1	1				
[LPE 18:0 + H]+		1	1			1	
[LPE 18:1 + H]+		1	1			1	
[LPE 20:1 + H]+			1				
[LPE 20:4 + H]+		1	1			1	

[LPE 22:4 + H]+		1	1				
[LPE 22:5 + H]+			1				
[LPE 22:6 + H]+		1	1			1	
[LPE O-16:1 + H]+			1				
[LPE O-18:1 + H]+			1				
Sum	*0*	*6*	*11*	*0*	*0*	*4*	*0*
**LPS**							
[LPS 18:0 + H]+			1				
[LPS 18:1 + H]+			1				
[LPS 22:1 + H]+			1				
[LPS 22:6 + H]+			1				
[LPS O-20:0 + H]+					1	1	
[LPS O-22:0;O + H]+						1	
Sum	*0*	*0*	*4*	*0*	*1*	*2*	*0*
**MG**							
[MG 16:0 + H-H_2O]+			1				
[MG 18:0 + H-H_2O]+		1	1				
[MG 18:1 + H-H_2O]+		1	1				
[MG 20:4 + H-H_2O]+			1				
[MG 22:6 + H-H_2O]+			1				
Sum	*0*	*2*	*5*	*0*	*0*	*0*	*0*
**PC**							
[PC 11:0/11:0 + H]+		1	1			1	
[PC 30:0 + H]+		1	1				
[PC 32:0 + H]+		1	1			1	
[PC 34:0 + H]+		1	1			1	
[PC 34:1 + H]+		1	1			1	
[PC 34:2 + H]+		1	1			1	
[PC 36:1 + H]+		1	1				
[PC 36:2 + H]+		1	1			1	
[PC 38:1 + H]+		1	1				
[PC 38:2 + H]+		1	1				
[PC 40:1 + H]+		1	1				
[PC 40:2 + H]+			1				
[PC 42:1 + H]+		1	1				
[PC 42:2 + H]+		1	1				
[PC O-32:0 + H]+			1				
[PC O-32:1 + H]+			1				
[PC O-32:2 + H]+			1				
[PC O-34:1 + H]+		1	1			1	
[PC O-34:2 + H]+		1	1				

[PC O-36:1 + H]+		1	1				
[PC O-36:2 + H]+		1	1				
Sum	*0*	*17*	*21*	*0*	*0*	*7*	*0*
**PE**							
[PE 34:0 + H]+			1				
[PE 34:1 + H]+		1	1				
[PE 36:0 + H]+			1				
[PE 36:1 + H]+		1	1				
[PE 36:2 + H]+		1	1				
[PE 38:1 + H]+		1	1				
[PE 40:4 + H]+		1	1				
[PE O-34:1 + H]+			1				
[PE O-34:2 + H]+		1	1			1	
[PE O-36:1 + H]+			1				
[PE O-36:2 + H]+		1	1			1	
[PE O-36:3 + H]+		1	1			1	
[PE O-38:2 + H]+			1				
[PE O-38:3 + H]+		1	1				
Sum	*0*	*9*	*14*	*0*	*0*	*3*	*0*
**PS**							
[PS 34:1 + H]+			1				
[PS 36:1 + H]+		1	1				
[PS 36:2 + H]+			1				
[PS 38:1 + H]+			1				
[PS 40:6 + H]+		1	1			1	
[PS 40:7 + H]+			1				
Sum	*0*	*2*	*6*	*0*	*0*	*1*	*0*
**SM**							
[SM 34:1;O_2 + H]+			1				
[SM 36:1;O_2 + H]+		1	1			1	
[SM 36:2;O_2 + H]+		1	1				
[SM 38:1;O_2 + H]+		1	1				
[SM 40:1;O_2 + H]+			1				
[SM 42:2;O_2 + H]+		1	1			1	
Sum	*0*	*4*	*6*	*0*	*0*	*2*	*0*
**SPB**							
[SPB 16:0;O_2 + H]+	1	1	1			1	
[SPB 17:0;O_2 + H]+		1				1	
[SPB 18:0;O_2 + H]+		1	1				
[SPB 20:1;O_2 + H-H_2O]+			1				

Sum	*1*	*3*	*3*	*0*	*0*	*2*	*0*
**Steroids**							
[ST 27:1;O + H-H_2O]+		1	1		1	1	
[ST 29:1;O + H-H_2O]+					1		
Sum	*0*	*1*	*1*	*0*	*2*	*1*	*0*

Table S5. Analytes detected in negative mode from standard solution and in RBE analyzed with the different LLE methods. The analytes were putatively annotated based on mass accuracy (< 5 ppm) and are divided in subcategories.

	BD up	BD low	BUME up	BUME low	PLE up	PLE mid	PLE low
**Essential amino acids**							
[15N-Ala - H]-	1			1			1
[15N-Asn - H]-	1			1			1
[15N-Asp - H]-	1		1	1			1
[15N-Gln - H]-	1			1			1
[15N-Glu - H]-	1		1	1			1
[15N-Gly - H]-				1			1
[15N- His - H]-	1			1			
[15N-Iso - H]-	1	1	1	1			1
[15N-Lys - H]-				1			1
[15N-Met - H]-				1			1
[15N-Phe - H]-	1	1	1	1			1
[15N-Pro - H]-				1			1
[15N-Ser - H]-	1			1			1
[15N-Thr - H]-	1		1	1			1
[15N-Try - H]-	1		1	1			1
[15N-Tyr - H]-	1			1			1
[15N-Val - H]-			1	1			1
[Ala - H]-	1	1	1	1		1	1
[Asp - H]-	1		1	1			1
Sum	*14*	*3*	*9*	*19*	*0*	*1*	*18*
**Other small metabolites**							
[Creatine - H]-	1			1			1
[Cystathionine - H - H_2O]-				1			
[Cystine - H - H_2O]-				1			
[Glucose - H]-				1			1
[Fumarate + Cl]-							
[Fumarate - H]-	1	1	1				

[GABA + Cl]-	1						1
[Glucose 6-phosphate - H]-				1			1
[Glycerate - H]-	1						
[GPEA- H]-	1			1			1
[Glycolate - H]-	1		1	1			
[Hydroxyproline - H]-	1	1	1	1		1	1
[Hypoxanthine - H]-	1	1	1	1			1
[Inosine - H]-	1			1			1
[Lactate - H]-		1	1	1	1		
[Malate - H]-	1	1	1	1		1	
[N-acetylaspartate - H]-	1	1	1	1		1	1
[Nicotinamide - H - H_2O]-			1				
[Pyruglutamate - H]-			1	1			
[Taurine - H]-	1		1	1			1
[Threonate - H]-	1		1	1		1	1
[Xanthine - H]-	1	1	1	1			1
Sum	*14*	*7*	*12*	*17*	*1*	*4*	*12*
**Fatty acyls**							
[D9-FA 18:1 - H]-			1				
[FA 3:1;O_2 - H]-	1						1
[FA 4:0;O_2 - H]-	1		1				
[FA 4:2;O_3 - H]-							1
[FA 4:1 - H]-							
[FA 4:1;O - H]-		1					
[FA 4:1;O_2 - H]-	1						
[FA 5:0 - H]-							1
[FA 5:0;O - H]-	1	1					
[FA 5:1 - H]-	1						
[FA 5:1;O_2 - H]-	1						
[FA 5:1;O_3 - H]-			1				
[FA 5:2 - H]-							
[FA 5:2;O - H]-	1						
[FA 5:2;O_2 - H]-	1			1			
[FA 6:0 - H]-				1			
[FA 6:1 - H]-	1						
[FA 6:1;O_2 - H]-			1				
[FA 6:2;O_2 - H]-	1						
[FA 6:2;O_3 - H]-							
[FA 6:2;O_4 - H]-							1
[FA 6:2;O_5 - H]-							
[FA 6:3;O_2 - H]-	1						
[FA 6:3;O_3 - H]-							
[FA 7:0 - H]-				1			

[FA 8:0 - H]-				1			
[FA 8:0;O_2 - H]-							
[FA 8:1 - H]-							1
[FA 8:1;O_3 - H]-			1				1
[FA 8:2 - H]-				1			1
[FA 8:2;O_2 - H]-				1			
[FA 8:3;O - H]-			1				
[FA 10:0 - H]-				1			
[FA 10:0;O_2 - H]-				1			
[FA 10:1;O_2 - H]-			1	1			
[FA 10:2 - H]-							
[FA 10:3 - H]-	1						
[FA 10:3;O - H]-							1
[FA 10:3;O_2 - H]-							1
[FA 10:3;O_3 - H]-			1				1
[FA 10:4 - H]-		1					
[FA 10:5;O - H]-	1						
[FA 10:5;O_2 - H]-			1				
[FA 12:1;O_4 - H]-			1				
[FA 12:2 - H]-							
[FA 12:2;O - H]-		1					
[FA 12:2;O_2 - H]-			1				
[FA 12:2;O_3 - H]-			1				
[FA 12:3 - H]-							1
[FA 12:3;O - H]-							
[FA 12:3;O_2 - H]-							
[FA 12:4 - H]-							1
[FA 12:4;O - H]-							1
[FA 12:4;O_3 - H]-			1				1
[FA 12:5;O_2 - H]-							
[FA 14:0;O - H]-			1				
[FA 14:1;O - H]-			1				
[FA 14:1;O_3 - H]-			1				
[FA 14:2;O - H]-							
[FA 14:3 - H]-							1
[FA 14:3;O - H]-							1
[FA 16:0 - H]-			1				
[FA 16:0;O_2 - H]-			1				
[FA 16:1 - H]-	1	1	1	1		1	1
[FA 16:1;O_2 - H]-			1				
[FA 16:1;O_3 - H]-			1				
[FA 16:2;O_2 - H]-			1				
[FA 16:3;O_2 - H]-			1				
[FA 16:3;O_3 - H]-			1				

[FA 18:0 - H]-			1				
[FA 18:0;O - H]-			1				
[FA 18:0;O_4 - H]-			1			1	
[FA 18:1 - H]-	1	1	1		1	1	1
[FA 18:1;O - H]-							
[FA 18:1;O_2 - H]-		1	1			1	
[FA 18:1;O_3 - H]-			1			1	
[FA 18:2 - H]-		1	1				
[FA 18:2;O - H]-						1	
[FA 18:2;O_2 - H]-		1	1				
[FA 18:2;O_3 - H]-			1				
[FA 18:3 - H]-		1	1			1	
[FA 18:3;O_4 - H]-		1					
[FA 20:1 - H]-			1				
[FA 20:1;O_3 - H]-			1				
[FA 20:4 - H]-		1	1			1	
[FA 22:4 - H]-			1				
[FA 22:6 - H]-		1	1			1	
[FAL 6:0 - H]-							1
[FAL 6:2 - H]-							
[FAL 8:3 - H]-							1
[FAL 14:3 - H]-							1
Sum	*15*	*13*	*39*	*10*	*1*	*9*	*21*
**LPA**							
[LPA 18:0 - H]-					1		
[LPA 18:1 - H]-		1					
[LPA O-18:0 - H]-						1	
[LPA O-20:1 + Cl]-		1					
Sum	*0*	*2*	*0*	*0*	*1*	*1*	*0*
**LPC**							
[LPC 18:1 + HCOO]-		1					
[LPC 20:4 + HCOO]-		1					
Sum	*0*	*2*	*0*	*0*	*0*	*0*	*0*
**LPE**							
[LPE 16:0 - H]-		1	1			1	
[LPE 18:0 - H]-		1	1			1	
[LPE 18:1 - H]-		1	1			1	
[LPE 20:1 - H]-		1	1			1	
[LPE 20:4 - H]-		1	1			1	
[LPE 22:4 - H]-		1	1			1	
[LPE 22:5 - H]-		1					

[LPE 22:6 - H]-		1	1			1	
[LPE O-16:1 - H]-		1	1				
[LPE O-18:1 - H]-		1	1			1	
Sum	*0*	*10*	*9*	*0*	*0*	*8*	*0*
**LPG**							
[LPG 18:1 - H]-		1					
[LPG 22:6 - H]-		1					
Sum	*0*	*2*	*0*	*0*	*0*	*0*	*0*
**LPI**							
[LPI 18:0 - H]-		1	1			1	
[LPI 20:4 - H]-		1	1				
Sum	*0*	*2*	*2*	*0*	*0*	*1*	*0*
**LPS**							
[LPS 18:0 - H]-		1	1			1	
[LPS 18:1 - H]-		1					
[LPS 20:4 - H]-		1					
[LPS 22:4 - H]-		1					
[LPS 22:6 - H]-		1	1			1	
Sum	*0*	*5*	*2*	*0*	*0*	*2*	*0*
**MG**							
[MG 18:1 - H]-			1			1	
[MG 22:1 - H]-			1		1	1	
Sum	*0*	*0*	*2*	*0*	*1*	*2*	*0*
**PA**							
[PA 34:1 - H]-		1					
[PA 36:1 - H]-		1	1				
[PA 36:2 - H]-		1					
[PA 38:4 - H]-		1					
Sum	*0*	*4*	*1*	*0*	*0*	*0*	*0*
**PC**							
[PC 11:0/11:0 + HCOO]-		1	1			1	
[PC 32:0 + HCOO]-		1	1			1	
[PC 34:1 + HCOO]-		1	1			1	
[PC 36:1 + HCOO]-		1	1				
Sum	*0*	*4*	*4*	*0*	*0*	*3*	*0*
**PE**							
[PE 34:1 - H]-			1			1	

[PE 36:1 - H]-		1	1			1	
[PE 36:2 - H]-			1			1	
[PE 36:4 - H]-			1				
[PE 38:4 - H]-			1			1	
[PE 40:4 - H]-			1			1	
[PE O-34:1 - H]-			1				
[PE O-34:2 - H]-		1	1			1	
[PE O-36:2 - H]-		1	1			1	
[PE O-36:3 - H]-		1	1			1	
[PE O-38:2 - H]-			1				
[PE O-38:3 - H]-			1			1	
Sum	*0*	*4*	*12*	*0*	*0*	*9*	*0*
**PG**							
[PG 32:0 - H]-		1					
[PG 34:1 - H]-		1					
[PG 36:2 - H]-		1					
[PG 36:4 - H]-		1					
[PG 38:4 - H]-		1					
Sum	*0*	*5*	*0*	*0*	*0*	*0*	*0*
**PI**							
[PI 36:1 - H]-		1					
[PI 36:2 - H]-		1					
[PI 36:4 - H]-		1	1			1	
[PI 38:4 - H]-		1	1			1	
[PI 38:5 - H]-		1	1			1	
[PI 38:6 - H]-		1					
[PI 40:6 - H]-		1					
Sum	*0*	*7*	*3*	*0*	*0*	*3*	*0*
**PS**							
[PS 36:1 - H]-		1	1			1	
[PS 36:2 - H]-		1	1			1	
[PS 38:1 - H]-		1					
[PS 38:4 - H]-		1	1			1	
[PS 40:4 - H]-		1	1			1	
Sum	*0*	*5*	*4*	*0*	*0*	*4*	*0*
**SHexCer**							
[SHexCer 34:1;O_2 - H]-		1					
[SHexCer 36:1;O_2 - H]-		1	1			1	
[SHexCer 38:1;O_2 - H]-		1					
[SHexCer 38:1;O_3 - H]-		1					

[SHexCer 40:1;O_2 - H]-		1	1				
[SHexCer 40:1;O_3 - H]-		1	1			1	
[SHexCer 42:1;O_2 - H]-		1	1			1	
[SHexCer 42:1;O_3 - H]-		1	1			1	
[SHexCer 42:2;O_2 - H]-		1	1			1	
[SHexCer 42:2;O_3 - H]-		1	1			1	
Sum	*0*	*10*	*7*	*0*	*0*	*6*	*0*

Table S6. Log P values of analytes and the corresponding extraction phase in the BUME extraction. All log P values were retrieved from The Human Metabolome Database (HMDB).

Name	log P	Extraction Phase
**Choline**	-3,6	Aqueous Phase
**Glucose**	-3,24	Aqueous Phase
**Glucose 6-phosphate**	-2,1	Aqueous Phase
**GABA**	-3,17	Aqueous Phase
**FA 16:0 (Palmitic acid)**	7,17	Organic Phase
**FA 20:4 (Arachidonic acid)**	6,98	Organic Phase
**LPE 16:0 (LysoPE(0:0/16:0))**	4,06	Organic Phase
**LPC 16:0 (LysoPC(0:0/16:0))**	1,82	Organic Phase
**LPC 20:1 (LysoPC(20:1(11Z)/0:0))**	2,99	Organic Phase
**PA 36:1 (PA(18:1(9Z)/18:0))**	9,27	Organic Phase
**PC 36:1 (PC(18:0/18:1(11Z)))**	5,88	Organic Phase
**PC 34:1 (PC(16:0/18:1(11Z)))**	5,58	Organic Phase
**PE 34:0 (PE(16:0/18:0))**	8,55	Organic Phase
**PE 38:1 (PE(18:0/20:1(11Z)))**	9,15	Organic Phase
**PS 34:1 (PS(16:0/18:1(11Z)))**	4,63	Organic Phase
**PS 36:1 (PS(18:0/18:1(9Z)))**	4,95	Organic Phase

Table S7. Analytes detected from INS-1 cell extraction in different LLE phases and in the one solvent method. Data collected in positive and normalized to either IS or TIC, as mentioned in the table. The correction factor used to make the bar graphs of figures 4 and S1 are detailed in the last column.

Analyte	Sampling	Relative signal Average	Standard deviation	Correction factor for graphs
**IS normalization**				
**[Glu +Na]+**	MeOH:H_2O	1.86E-02	6.36E-03	*50
**[Glu +Na]+**	LLE Lower	2.58E-02	1.22E-02	*50
**[Glu +Na]+**	LLE upper	0.00E+00	0.00E+00	*50
**[Glucose +K]+**	MeOH:H_2O	1.62E+00	9.94E-02	
**[Glucose +K]+**	LLE Lower	1.68E+00	1.11E-01	
**[Glucose +K]+**	LLE upper	0.00E+00	0.00E+00	
**[Glucose +Na]+**	MeOH:H_2O	1.54E+00	1.43E-01	
**[Glucose +Na]+**	LLE Lower	1.44E+00	7.46E-02	
**[Glucose +Na]+**	LLE upper	0.00E+00	0.00E+00	
**[Pro +H]+**	MeOH:H_2O	1.72E-02	2.90E-03	*50
**[Pro +H]+**	LLE Lower	1.27E-02	2.53E-04	*50
**[Pro +H]+**	LLE upper	2.98E-02	1.58E-02	*50
**[Val +H]+**	MeOH:H_2O	3.05E-01	2.07E-02	*10
**[Val +H]+**	LLE Lower	1.78E-01	3.04E-02	*10
**[Val +H]+**	LLE upper	2.85E-01	1.10E-02	*10
**[Val +Na]+**	MeOH:H_2O	1.08E+00	1.83E-01	
**[Val +Na]+**	LLE Lower	3.78E+00	1.67E+00	
**[Val +Na]+**	LLE upper	0.00E+00	0.00E+00	
**[PC 30:0 +H]+**	MeOH:H_2O	8.03E-03	2.32E-04	
**[PC 30:0 +H]+**	LLE Lower	0.00E+00	0.00E+00	
**[PC 30:0 +H]+**	LLE upper	9.54E-03	1.00E-04	
**[PC 32:0 +H]+**	MeOH:H_2O	1.74E-02	3.68E-04	
**[PC 32:0 +H]+**	LLE Lower	0.00E+00	0.00E+00	
**[PC 32:0 +H]+**	LLE upper	2.08E-02	7.64E-04	
**[PC 32:1 +H]+**	MeOH:H_2O	3.90E-02	6.60E-04	
**[PC 32:1 +H]+**	LLE Lower	0.00E+00	0.00E+00	
**[PC 32:1 +H]+**	LLE upper	3.84E-02	1.45E-03	
**[PC 32:2 +H]+**	MeOH:H_2O	3.03E-03	4.63E-04	*10
**[PC 32:2 +H]+**	LLE Lower	0.00E+00	0.00E+00	*10
**[PC 32:2 +H]+**	LLE upper	3.31E-03	1.55E-04	*10
**[PC 34:1 +H]+**	MeOH:H_2O	1.45E-01	4.05E-03	/5
**[PC 34:1 +H]+**	LLE Lower	0.00E+00	0.00E+00	/5
**[PC 34:1 +H]+**	LLE upper	1.36E-01	3.09E-03	/5
**[PC 34:2 +H]+**	MeOH:H_2O	4.37E-02	1.40E-03	
**[PC 34:2 +H]+**	LLE Lower	0.00E+00	0.00E+00	

**[PC 34:2 +H]+**	LLE upper	3.88E-02	2.30E-03	
**[PC 36:1 +H]+**	MeOH:H_2O	2.90E-02	5.48E-04	
**[PC 36:1 +H]+**	LLE Lower	0.00E+00	0.00E+00	
**[PC 36:1 +H]+**	LLE upper	2.68E-02	3.87E-04	
**[PC 36:2 +H]+**	MeOH:H_2O	8.41E-02	2.79E-03	/2
**[PC 36:2 +H]+**	LLE Lower	0.00E+00	0.00E+00	/2
**[PC 36:2 +H]+**	LLE upper	6.99E-02	2.57E-03	/2
**[PC 38:2 +H]+**	MeOH:H_2O	4.48E-03	1.84E-04	*10
**[PC 38:2 +H]+**	LLE Lower	0.00E+00	0.00E+00	*10
**[PC 38:2 +H]+**	LLE upper	3.60E-03	7.21E-05	*10
**[PC O-32:1 +H]+**	MeOH:H_2O	0.00E+00	0.00E+00	*10
**[PC O-32:1 +H]+**	LLE Lower	0.00E+00	0.00E+00	*10
**[PC O-32:1 +H]+**	LLE upper	1.00E-03	3.68E-04	*10
**[PC O-34:1 +H]+**	MeOH:H_2O	5.52E-03	4.39E-04	*4
**[PC O-34:1 +H]+**	LLE Lower	0.00E+00	0.00E+00	*4
**[PC O-34:1 +H]+**	LLE upper	6.43E-03	1.62E-04	*4
**TIC normalization**				
**[Creatine +H]+**	MeOH:H_2O	1.81E-03	2.42E-04	/4
**[Creatine +H]+**	LLE Lower	1.29E-03	5.49E-04	/4
**[Creatine +H]+**	LLE upper	5.56E-05	4.02E-05	/4
**[GPC +H]+**	MeOH:H_2O	3.29E-04	3.95E-05	
**[GPC +H]+**	LLE Lower	2.25E-04	8.27E-05	
**[GPC +H]+**	LLE upper	1.92E-05	1.44E-05	
**[GPC +Na]+**	MeOH:H_2O	7.90E-04	1.08E-04	
**[GPC +Na]+**	LLE Lower	4.44E-04	1.92E-04	
**[GPC +Na]+**	LLE upper	0.00E+00	0.00E+00	
**[Phe +H]+**	MeOH:H_2O	0.00E+00	0.00E+00	*4
**[Phe +H]+**	LLE Lower	1.06E-04	2.40E-05	*4
**[Phe +H]+**	LLE upper	0.00E+00	0.00E+00	*4
**[Pyruglutamate +H]+**	MeOH:H_2O	5.41E-05	2.15E-05	*4
**[Pyruglutamate +H]+**	LLE Lower	9.46E-05	3.72E-06	*4
**[Pyruglutamate +H]+**	LLE upper	0.00E+00	0.00E+00	*4
**[Taurine +H]+**	MeOH:H_2O	0.00E+00	0.00E+00	*4
**[Taurine +H]+**	LLE Lower	1.20E-04	2.54E-06	*4
**[Taurine +H]+**	LLE upper	0.00E+00	0.00E+00	*4
**[Taurine +Na]+**	MeOH:H_2O	2.99E-04	3.28E-05	
**[Taurine +Na]+**	LLE Lower	3.77E-04	8.21E-05	
**[Taurine +Na]+**	LLE upper	0.00E+00	0.00E+00	
**[FA 5:0 +H]+**	MeOH:H_2O	0.00E+00	0.00E+00	
**[FA 5:0 +H]+**	LLE Lower	0.00E+00	0.00E+00	
**[FA 5:0 +H]+**	LLE upper	2.13E-04	2.30E-05	
**[FA 6:0 +H]+**	MeOH:H_2O	0.00E+00	0.00E+00	/4
**[FA 6:0 +H]+**	LLE Lower	0.00E+00	0.00E+00	/4
**[FA 6:0 +H]+**	LLE upper	8.37E-04	8.37E-05	/4

**[FA 6:1 +H]+**	MeOH:H_2O	0.00E+00	0.00E+00	
**[FA 6:1 +H]+**	LLE Lower	0.00E+00	0.00E+00	
**[FA 6:1 +H]+**	LLE upper	8.93E-05	7.17E-06	
**[FA 6:2 +H]+**	MeOH:H_2O	0.00E+00	0.00E+00	
**[FA 6:2 +H]+**	LLE Lower	0.00E+00	0.00E+00	
**[FA 6:2 +H]+**	LLE upper	8.81E-05	4.77E-06	
**[FA 8:0 +H]+**	MeOH:H_2O	0.00E+00	0.00E+00	/100
**[FA 8:0 +H]+**	LLE Lower	0.00E+00	0.00E+00	/100
**[FA 8:0 +H]+**	LLE upper	3.76E-03	2.61E-04	/100
**[FA 8:0;O +H]+**	MeOH:H_2O	0.00E+00	0.00E+00	/100
**[FA 8:0;O +H]+**	LLE Lower	0.00E+00	0.00E+00	/100
**[FA 8:0;O +H]+**	LLE upper	2.25E-02	1.48E-03	/100
**[FA 8:0;O +H-H_2O]+**	MeOH:H_2O	0.00E+00	0.00E+00	/10
**[FA 8:0;O +H-H_2O]+**	LLE Lower	0.00E+00	0.00E+00	/10
**[FA 8:0;O +H-H_2O]+**	LLE upper	1.23E-03	1.14E-04	/10
**[FA 8:0;O_2 +H]+**	MeOH:H_2O	0.00E+00	0.00E+00	/4
**[FA 8:0;O_2 +H]+**	LLE Lower	0.00E+00	0.00E+00	/4
**[FA 8:0;O_2 +H]+**	LLE upper	9.45E-04	7.21E-05	/4
**[FA 8:2 +H]+**	MeOH:H_2O	0.00E+00	0.00E+00	
**[FA 8:2 +H]+**	LLE Lower	0.00E+00	0.00E+00	
**[FA 8:2 +H]+**	LLE upper	1.08E-04	6.45E-06	
**[FA 8:2;O +H-H_2O]+**	MeOH:H_2O	0.00E+00	0.00E+00	
**[FA 8:2;O +H-H_2O]+**	LLE Lower	0.00E+00	0.00E+00	
**[FA 8:2;O +H-H_2O]+**	LLE upper	3.17E-05	1.11E-05	
**[FA 8:3;O +H-H_2O]+**	MeOH:H_2O	0.00E+00	0.00E+00	
**[FA 8:3;O +H-H_2O]+**	LLE Lower	0.00E+00	0.00E+00	
**[FA 8:3;O +H-H_2O]+**	LLE upper	5.15E-05	7.96E-06	
**[FA 10:0;O +H]+**	MeOH:H_2O	0.00E+00	0.00E+00	
**[FA 10:0;O +H]+**	LLE Lower	0.00E+00	0.00E+00	
**[FA 10:0;O +H]+**	LLE upper	2.87E-04	2.01E-05	
**[FA 10:1 +H]+**	MeOH:H_2O	0.00E+00	0.00E+00	
**[FA 10:1 +H]+**	LLE Lower	0.00E+00	0.00E+00	
**[FA 10:1 +H]+**	LLE upper	7.78E-05	4.23E-06	
**[FA 10:2 +H]+**	MeOH:H_2O	0.00E+00	0.00E+00	
**[FA 10:2 +H]+**	LLE Lower	0.00E+00	0.00E+00	
**[FA 10:2 +H]+**	LLE upper	1.72E-04	2.22E-05	
**[FA 10:3 +H]+**	MeOH:H_2O	0.00E+00	0.00E+00	
**[FA 10:3 +H]+**	LLE Lower	0.00E+00	0.00E+00	
**[FA 10:3 +H]+**	LLE upper	1.54E-04	1.68E-05	
**[FA 10:4 +H]+**	MeOH:H_2O	0.00E+00	0.00E+00	
**[FA 10:4 +H]+**	LLE Lower	0.00E+00	0.00E+00	
**[FA 10:4 +H]+**	LLE upper	3.40E-04	4.57E-05	
**[FA 10:4 +H-H_2O]+**	MeOH:H_2O	0.00E+00	0.00E+00	
**[FA 10:4 +H-H_2O]+**	LLE Lower	0.00E+00	0.00E+00	

**[FA 10:4 +H-H_2O]+**	LLE upper	1.16E-04	1.78E-05	
**[FA 12:0;O +H]+**	MeOH:H_2O	0.00E+00	0.00E+00	
**[FA 12:0;O +H]+**	LLE Lower	0.00E+00	0.00E+00	
**[FA 12:0;O +H]+**	LLE upper	3.46E-04	2.90E-05	
**[FA 12:2 +H-H_2O]+**	MeOH:H_2O	0.00E+00	0.00E+00	
**[FA 12:2 +H-H_2O]+**	LLE Lower	0.00E+00	0.00E+00	
**[FA 12:2 +H-H_2O]+**	LLE upper	6.46E-05	2.13E-05	
**[FA 12:2;O +H]+**	MeOH:H_2O	0.00E+00	0.00E+00	
**[FA 12:2;O +H]+**	LLE Lower	0.00E+00	0.00E+00	
**[FA 12:2;O +H]+**	LLE upper	7.39E-05	1.06E-05	
**[FA 12:3 +H]+**	MeOH:H_2O	0.00E+00	0.00E+00	
**[FA 12:3 +H]+**	LLE Lower	0.00E+00	0.00E+00	
**[FA 12:3 +H]+**	LLE upper	7.54E-05	4.04E-06	
**[FA 12:3 +H-H_2O]+**	MeOH:H_2O	0.00E+00	0.00E+00	
**[FA 12:3 +H-H_2O]+**	LLE Lower	0.00E+00	0.00E+00	
**[FA 12:3 +H-H_2O]+**	LLE upper	3.60E-05	1.63E-05	
**[FA 12:6;O +H]+**	MeOH:H_2O	0.00E+00	0.00E+00	
**[FA 12:6;O +H]+**	LLE Lower	0.00E+00	0.00E+00	
**[FA 12:6;O +H]+**	LLE upper	7.69E-05	6.90E-06	
**[FA 14:0 +H]+**	MeOH:H_2O	0.00E+00	0.00E+00	
**[FA 14:0 +H]+**	LLE Lower	0.00E+00	0.00E+00	
**[FA 14:0 +H]+**	LLE upper	1.09E-04	6.67E-06	
**[FA 14:3 +H]+**	MeOH:H_2O	0.00E+00	0.00E+00	
**[FA 14:3 +H]+**	LLE Lower	0.00E+00	0.00E+00	
**[FA 14:3 +H]+**	LLE upper	1.12E-04	8.39E-06	
**[FA 14:3;O_2 +H]+**	MeOH:H_2O	0.00E+00	0.00E+00	
**[FA 14:3;O_2 +H]+**	LLE Lower	0.00E+00	0.00E+00	
**[FA 14:3;O_2 +H]+**	LLE upper	4.50E-05	1.13E-05	
**[FA 14:4 +H]+**	MeOH:H_2O	0.00E+00	0.00E+00	
**[FA 14:4 +H]+**	LLE Lower	0.00E+00	0.00E+00	
**[FA 14:4 +H]+**	LLE upper	3.48E-04	4.25E-05	
**[FA 16:0 +H]+**	MeOH:H_2O	0.00E+00	0.00E+00	/2
**[FA 16:0 +H]+**	LLE Lower	0.00E+00	0.00E+00	/2
**[FA 16:0 +H]+**	LLE upper	5.51E-04	4.65E-05	/2
**[FA 16:1 +H]+**	MeOH:H_2O	0.00E+00	0.00E+00	
**[FA 16:1 +H]+**	LLE Lower	0.00E+00	0.00E+00	
**[FA 16:1 +H]+**	LLE upper	1.93E-04	2.51E-05	
**[FA 16:1;O +H]+**	MeOH:H_2O	0.00E+00	0.00E+00	
**[FA 16:1;O +H]+**	LLE Lower	0.00E+00	0.00E+00	
**[FA 16:1;O +H]+**	LLE upper	1.18E-04	1.35E-05	
**[FA 16:2 +H]+**	MeOH:H_2O	0.00E+00	0.00E+00	
**[FA 16:2 +H]+**	LLE Lower	0.00E+00	0.00E+00	
**[FA 16:2 +H]+**	LLE upper	1.91E-04	1.78E-05	
**[FA 16:2;O +H]+**	MeOH:H_2O	0.00E+00	0.00E+00	

**[FA 16:2;O +H]+**	LLE Lower	0.00E+00	0.00E+00	
**[FA 16:2;O +H]+**	LLE upper	3.91E-05	1.86E-05	
**[FA 18:0 +H]+**	MeOH:H_2O	0.00E+00	0.00E+00	
**[FA 18:0 +H]+**	LLE Lower	0.00E+00	0.00E+00	
**[FA 18:0 +H]+**	LLE upper	1.68E-04	1.57E-05	
**[FA 18:1;O +H]+**	MeOH:H_2O	0.00E+00	0.00E+00	
**[FA 18:1;O +H]+**	LLE Lower	0.00E+00	0.00E+00	
**[FA 18:1;O +H]+**	LLE upper	7.37E-05	9.54E-06	
**[FA 18:2 +H]+**	MeOH:H_2O	0.00E+00	0.00E+00	
**[FA 18:2 +H]+**	LLE Lower	0.00E+00	0.00E+00	
**[FA 18:2 +H]+**	LLE upper	1.28E-04	1.37E-05	
**[FA 18:3 +H]+**	MeOH:H_2O	0.00E+00	0.00E+00	
**[FA 18:3 +H]+**	LLE Lower	0.00E+00	0.00E+00	
**[FA 18:3 +H]+**	LLE upper	9.28E-05	3.28E-05	
**[FA 20:2 +H]+**	MeOH:H_2O	0.00E+00	0.00E+00	
**[FA 20:2 +H]+**	LLE Lower	0.00E+00	0.00E+00	
**[FA 20:2 +H]+**	LLE upper	8.63E-05	2.27E-05	
**[FA 22:1;O_2 +H]+**	MeOH:H_2O	0.00E+00	0.00E+00	
**[FA 22:1;O_2 +H]+**	LLE Lower	0.00E+00	0.00E+00	
**[FA 22:1;O_2 +H]+**	LLE upper	1.42E-04	2.04E-06	
**[FA 22:5;O_3 +H]+**	MeOH:H_2O	0.00E+00	0.00E+00	
**[FA 22:5;O_3 +H]+**	LLE Lower	0.00E+00	0.00E+00	
**[FA 22:5;O_3 +H]+**	LLE upper	9.07E-05	1.39E-05	
**[FAL 6:0 +H]+**	MeOH:H_2O	0.00E+00	0.00E+00	
**[FAL 6:0 +H]+**	LLE Lower	0.00E+00	0.00E+00	
**[FAL 6:0 +H]+**	LLE upper	1.31E-04	9.99E-06	
**[FAL 8:1 +H-H_2O]+**	MeOH:H_2O	0.00E+00	0.00E+00	/10
**[FAL 8:1 +H-H_2O]+**	LLE Lower	0.00E+00	0.00E+00	/10
**[FAL 8:1 +H-H_2O]+**	LLE upper	1.67E-03	1.16E-04	/10
**[FAL 8:2 +H]+**	MeOH:H_2O	0.00E+00	0.00E+00	
**[FAL 8:2 +H]+**	LLE Lower	0.00E+00	0.00E+00	
**[FAL 8:2 +H]+**	LLE upper	1.36E-04	1.46E-05	
**[FAL 8:2 +H-H_2O]+**	MeOH:H_2O	0.00E+00	0.00E+00	
**[FAL 8:2 +H-H_2O]+**	LLE Lower	0.00E+00	0.00E+00	
**[FAL 8:2 +H-H_2O]+**	LLE upper	1.90E-04	2.62E-05	
**[FAL 8:3 +H-H_2O]+**	MeOH:H_2O	0.00E+00	0.00E+00	
**[FAL 8:3 +H-H_2O]+**	LLE Lower	0.00E+00	0.00E+00	
**[FAL 8:3 +H-H_2O]+**	LLE upper	3.92E-04	2.51E-05	
**[FAL 10:2 +H]+**	MeOH:H_2O	0.00E+00	0.00E+00	
**[FAL 10:2 +H]+**	LLE Lower	0.00E+00	0.00E+00	
**[FAL 10:2 +H]+**	LLE upper	1.43E-04	1.77E-05	
**[FAL 10:2 +H-H_2O]+**	MeOH:H_2O	0.00E+00	0.00E+00	
**[FAL 10:2 +H-H_2O]+**	LLE Lower	0.00E+00	0.00E+00	
**[FAL 10:2 +H-H_2O]+**	LLE upper	2.41E-04	5.06E-06	

**[FAL 10:3 +H]+**	MeOH:H_2O	0.00E+00	0.00E+00	
**[FAL 10:3 +H]+**	LLE Lower	0.00E+00	0.00E+00	
**[FAL 10:3 +H]+**	LLE upper	4.30E-04	3.46E-05	
**[FAL 10:3 +H-H_2O]+**	MeOH:H_2O	0.00E+00	0.00E+00	/2
**[FAL 10:3 +H-H_2O]+**	LLE Lower	0.00E+00	0.00E+00	/2
**[FAL 10:3 +H-H_2O]+**	LLE upper	6.17E-04	8.87E-05	/2
**[FAL 10:4 +H]+**	MeOH:H_2O	0.00E+00	0.00E+00	
**[FAL 10:4 +H]+**	LLE Lower	0.00E+00	0.00E+00	
**[FAL 10:4 +H]+**	LLE upper	6.76E-05	1.14E-05	
**[FAL 10:4 +H-H_2O]+**	MeOH:H_2O	0.00E+00	0.00E+00	
**[FAL 10:4 +H-H_2O]+**	LLE Lower	0.00E+00	0.00E+00	
**[FAL 10:4 +H-H_2O]+**	LLE upper	5.44E-05	1.48E-05	
**[FAL 12:2 +H]+**	MeOH:H_2O	0.00E+00	0.00E+00	
**[FAL 12:2 +H]+**	LLE Lower	0.00E+00	0.00E+00	
**[FAL 12:2 +H]+**	LLE upper	1.13E-04	9.04E-06	
**[FAL 14:2 +H]+**	MeOH:H_2O	0.00E+00	0.00E+00	
**[FAL 14:2 +H]+**	LLE Lower	0.00E+00	0.00E+00	
**[FAL 14:2 +H]+**	LLE upper	6.23E-05	1.01E-05	
**[FAL 14:2 +H-H_2O]+**	MeOH:H_2O	0.00E+00	0.00E+00	
**[FAL 14:2 +H-H_2O]+**	LLE Lower	0.00E+00	0.00E+00	
**[FAL 14:2 +H-H_2O]+**	LLE upper	1.66E-04	2.28E-06	
**[FAL 14:3 +H]+**	MeOH:H_2O	0.00E+00	0.00E+00	
**[FAL 14:3 +H]+**	LLE Lower	0.00E+00	0.00E+00	
**[FAL 14:3 +H]+**	LLE upper	8.66E-05	1.86E-05	
**[FAL 14:4 +H]+**	MeOH_H2O	0.00E+00	0.00E+00	
**[FAL 14:4 +H]+**	LLE Lower	0.00E+00	0.00E+00	
**[FAL 14:4 +H]+**	LLE upper	1.29E-04	1.37E-05	
**[FAL 14:4 +H-H_2O]+**	MeOH:H_2O	0.00E+00	0.00E+00	
**[FAL 14:4 +H-H_2O]+**	LLE Lower	0.00E+00	0.00E+00	
**[FAL 14:4 +H-H_2O]+**	LLE upper	8.78E-05	2.78E-05	
**[FAL 16:3 +H]+**	MeOH:H_2O	0.00E+00	0.00E+00	
**[FAL 16:3 +H]+**	LLE Lower	0.00E+00	0.00E+00	
**[FAL 16:3 +H]+**	LLE upper	2.56E-04	1.08E-05	
**[FOH 6:0;O +H]+**	MeOH:H_2O	0.00E+00	0.00E+00	
**[FOH 6:0;O +H]+**	LLE Lower	0.00E+00	0.00E+00	
**[FOH 6:0;O +H]+**	LLE upper	6.18E-04	6.63E-05	
**[FOH 8:0 +H]+**	MeOH:H_2O	0.00E+00	0.00E+00	
**[FOH 8:0 +H]+**	LLE Lower	0.00E+00	0.00E+00	
**[FOH 8:0 +H]+**	LLE upper	4.03E-04	3.57E-05	
**[FOH 8:5 +H]+**	MeOH:H_2O	0.00E+00	0.00E+00	*10
**[FOH 8:5 +H]+**	LLE Lower	0.00E+00	0.00E+00	*10
**[FOH 8:5 +H]+**	LLE upper	5.45E-05	8.41E-06	*10
**[FOH 10:0;O +H]+**	MeOH:H_2O	0.00E+00	0.00E+00	/5
**[FOH 10:0;O +H]+**	LLE Lower	0.00E+00	0.00E+00	/5

**[FOH 10:0;O +H]+**	LLE upper	2.14E-03	1.66E-04	/5
**[FOH 12:3 +H-H_2O]+**	MeOH:H_2O	0.00E+00	0.00E+00	
**[FOH 12:3 +H-H_2O]+**	LLE Lower	0.00E+00	0.00E+00	
**[FOH 12:3 +H-H_2O]+**	LLE upper	1.94E-04	2.45E-05	
**[MG 16:0 +H-H_2O]+**	MeOH:H_2O	0.00E+00	0.00E+00	
**[MG 16:0 +H-H_2O]+**	LLE Lower	0.00E+00	0.00E+00	
**[MG 16:0 +H-H_2O]+**	LLE upper	1.32E-04	2.93E-05	
**[MG;O-12:3 +H-H_2O]+**	MeOH:H_2O	0.00E+00	0.00E+00	
**[MG;O-12:3 +H-H_2O]+**	LLE Lower	0.00E+00	0.00E+00	
**[MG;O-12:3 +H-H_2O]+**	LLE upper	4.53E-05	1.85E-05	
**[MG;O-12:4 +H-H_2O]+**	MeOH:H_2O	0.00E+00	0.00E+00	
**[MG;O-12:4 +H-H_2O]+**	LLE Lower	0.00E+00	0.00E+00	
**[MG;O-12:4 +H-H_2O]+**	LLE upper	2.16E-04	3.47E-05	
**[MG;O-16:0 +H-H_2O]+**	MeOH:H_2O	0.00E+00	0.00E+00	
**[MG;O-16:0 +H-H_2O]+**	LLE Lower	0.00E+00	0.00E+00	
**[MG;O-16:0 +H-H_2O]+**	LLE upper	1.03E-04	2.66E-05	
**[PE 36:1 +H]+**	MeOH:H_2O	1.63E-04	1.75E-05	
**[PE 36:1 +H]+**	LLE Lower	0.00E+00	0.00E+00	
**[PE 36:1 +H]+**	LLE upper	2.44E-04	1.12E-05	
**[PE 36:2 +H]+**	MeOH:H_2O	9.74E-05	3.02E-06	
**[PE 36:2 +H]+**	LLE Lower	0.00E+00	0.00E+00	
**[PE 36:2 +H]+**	LLE upper	1.52E-04	9.51E-06	
**[PE 38:1 +H]+**	MeOH:H_2O	4.87E-05	3.07E-05	
**[PE 38:1 +H]+**	LLE Lower	0.00E+00	0.00E+00	
**[PE 38:1 +H]+**	LLE upper	8.46E-05	2.74E-06	
**[PE 38:2 +H]+**	MeOH:H_2O	5.05E-05	7.51E-06	
**[PE 38:2 +H]+**	LLE Lower	0.00E+00	0.00E+00	
**[PE 38:2 +H]+**	LLE upper	9.20E-05	3.28E-05	
**[SM 34:1;O_2 +H]+**	MeOH:H_2O	2.44E-04	1.26E-05	
**[SM 34:1;O_2 +H]+**	LLE Lower	0.00E+00	0.00E+00	
**[SM 34:1;O_2 +H]+**	LLE upper	4.26E-04	4.64E-06	
**[SM 36:1;O_2 +H]+**	MeOH:H_2O	1.33E-04	8.02E-06	
**[SM 36:1;O_2 +H]+**	LLE Lower	0.00E+00	0.00E+00	
**[SM 36:1;O_2 +H]+**	LLE upper	2.44E-04	8.51E-06	
**[SPB 16:0;O_2 +H-H_2O]+**	MeOH:H_2O	0.00E+00	0.00E+00	
**[SPB 16:0;O_2 +H-H_2O]+**	LLE Lower	0.00E+00	0.00E+00	
**[SPB 16:0;O_2 +H-H_2O]+**	LLE upper	1.99E-04	1.18E-05	
**[SPB 16:1;O_2 +H-H_2O]+**	MeOH:H_2O	0.00E+00	0.00E+00	
**[SPB 16:1;O_2 +H-H_2O]+**	LLE Lower	0.00E+00	0.00E+00	
**[SPB 16:1;O_2 +H-H_2O]+**	LLE upper	7.59E-05	1.74E-06	
**[SPB 18:0;O_2 +H-H_2O]+**	MeOH:H_2O	0.00E+00	0.00E+00	
**[SPB 18:0;O_2 +H-H_2O]+**	LLE Lower	0.00E+00	0.00E+00	
**[SPB 18:0;O_2 +H-H_2O]+**	LLE upper	1.50E-04	9.27E-07	
**[SPB 18:1;O_2 +H-H_2O]+**	MeOH:H_2O	0.00E+00	0.00E+00	

**[SPB 18:1;O_2 +H-H_2O]+**	LLE Lower	0.00E+00	0.00E+00	
**[SPB 18:1;O_2 +H-H_2O]+**	LLE upper	1.06E-04	1.73E-05	
**[CAR 16:0 +H]+**	MeOH:H_2O	0.00E+00	0.00E+00	
**[CAR 16:0 +H]+**	LLE Lower	0.00E+00	0.00E+00	
**[CAR 16:0 +H]+**	LLE upper	1.51E-04	3.63E-05	
**[CAR 18:1 +H]+**	MeOH:H_2O	0.00E+00	0.00E+00	
**[CAR 18:1 +H]+**	LLE Lower	0.00E+00	0.00E+00	
**[CAR 18:1 +H]+**	LLE upper	8.42E-05	1.30E-05	
**[ST 27:2;O +H]+**	MeOH:H_2O	0.00E+00	0.00E+00	
**[ST 27:2;O +H]+**	LLE Lower	0.00E+00	0.00E+00	
**[ST 27:2;O +H]+**	LLE upper	1.52E-04	2.24E-05	
**[ST 27:2;O +H-H_2O]+**	MeOH:H_2O	0.00E+00	0.00E+00	
**[ST 27:2;O +H-H_2O]+**	LLE Lower	0.00E+00	0.00E+00	
**[ST 27:2;O +H-H_2O]+**	LLE upper	7.69E-05	1.66E-05	

Table S8. Analytes detected from INS-1 cell extraction in different LLE phases and in the one solvent method. Data collected in negative and normalized to either IS or TIC, as mentioned in the table. The correction factor used to make the bar graphs of figures 4 and S2 are detailed in the last column.

Analyte	Sampling	Relative signal Average	Standard deviation	Correction factor for graphs
**IS normalization**				
**[Ala - H]-**	MeOH:H_2O	2.83E-03	5.87E-04	*4
**[Ala - H]-**	LLE Lower	1.04E-02	1.57E-03	*4
**[Ala - H]-**	LLE upper	0.00E+00	0.00E+00	*4
**[Glu - H]-**	MeOH:H_2O	7.03E-02	3.18E-03	/2
**[Glu - H]-**	LLE Lower	6.05E-02	3.28E-03	/2
**[Glu - H]-**	LLE upper	0.00E+00	0.00E+00	/2
**[Glucose - H]-**	MeOH:H_2O	2.85E+00	2.50E-01	/100
**[Glucose - H]-**	LLE Lower	2.09E+00	1.73E-01	/100
**[Glucose - H]-**	LLE upper	0.00E+00	0.00E+00	/100
**[PG 34:1 - H]-**	MeOH:H_2O	0.00E+00	0.00E+00	
**[PG 34:1 - H]-**	LLE Lower	0.00E+00	0.00E+00	
**[PG 34:1 - H]-**	LLE upper	2.42E-02	2.79E-03	
**[PG 34:2 - H]-**	MeOH:H_2O	0.00E+00	0.00E+00	
**[PG 34:2 - H]-**	LLE Lower	0.00E+00	0.00E+00	
**[PG 34:2 - H]-**	LLE upper	1.02E-02	8.98E-04	
**[PG 36:1 - H]-**	MeOH:H_2O	0.00E+00	0.00E+00	
**[PG 36:1 - H]-**	LLE Lower	0.00E+00	0.00E+00	
**[PG 36:1 - H]-**	LLE upper	1.21E-02	1.17E-03	
**[PG 36:2 - H]-**	MeOH:H_2O	0.00E+00	0.00E+00	
**[PG 36:2 - H]-**	LLE Lower	0.00E+00	0.00E+00	
**[PG 36:2 - H]-**	LLE upper	3.20E-02	3.60E-03	
**[PG 36:3 - H]-**	MeOH:H_2O	0.00E+00	0.00E+00	*5
**[PG 36:3 - H]-**	LLE Lower	0.00E+00	0.00E+00	*5
**[PG 36:3 - H]-**	LLE upper	4.39E-03	1.87E-03	*5
**[PG 40:7 - H]-**	MeOH:H_2O	0.00E+00	0.00E+00	*5
**[PG 40:7 - H]-**	LLE Lower	0.00E+00	0.00E+00	*5
**[PG 40:7 - H]-**	LLE upper	3.10E-03	1.01E-03	*5
**TIC normalization**				
**[FA 8:2 + HCOO]-**	MeOH:H_2O	0.00E+00	0.00E+00	
**[FA 8:2 + HCOO]-**	LLE Lower	0.00E+00	0.00E+00	
**[FA 8:2 + HCOO]-**	LLE upper	8.89E-04	2.10E-04	
**[FA 8:2;O_2 - H]-**	MeOH:H_2O	5.69E-05	1.11E-05	
**[FA 8:2;O_2 - H]-**	LLE Lower	0.00E+00	0.00E+00	
**[FA 8:2;O_2 - H]-**	LLE upper	1.13E-03	1.15E-04	
**[FA 10:0;O_2 - H- H_2O]-**	MeOH:H_2O	1.39E-04	1.24E-05	
**[FA 10:0;O_2 - H- H_2O]-**	LLE Lower	0.00E+00	0.00E+00	
**[FA 10:0;O_2 - H- H_2O]-**	LLE upper	7.57E-04	1.12E-04	

**[FA 10:1;O_3 - H- H_2O]-**	MeOH:H_2O	1.15E-04	6.31E-05	
**[FA 10:1;O_3 - H- H_2O]-**	LLE Lower	0.00E+00	0.00E+00	
**[FA 10:1;O_3 - H- H_2O]-**	LLE upper	3.67E-04	2.88E-05	
**[FA 10:3;O_3 - H- H_2O]-**	MeOH:H_2O	0.00E+00	0.00E+00	
**[FA 10:3;O_3 - H- H_2O]-**	LLE Lower	0.00E+00	0.00E+00	
**[FA 10:3;O_3 - H- H_2O]-**	LLE upper	2.43E-04	5.06E-05	
**[FA 12:1 - H]-**	MeOH:H_2O	0.00E+00	0.00E+00	*4
**[FA 12:1 - H]-**	LLE Lower	0.00E+00	0.00E+00	*4
**[FA 12:1 - H]-**	LLE upper	1.02E-04	1.27E-05	*4
**[FA 12:1;O - H]-**	MeOH:H_2O	0.00E+00	0.00E+00	
**[FA 12:1;O - H]-**	LLE Lower	0.00E+00	0.00E+00	
**[FA 12:1;O - H]-**	LLE upper	4.71E-04	6.52E-05	
**[FA 12:2 + HCOO]-**	MeOH:H_2O	0.00E+00	0.00E+00	
**[FA 12:2 + HCOO]-**	LLE Lower	0.00E+00	0.00E+00	
**[FA 12:2 + HCOO]-**	LLE upper	3.06E-04	3.18E-05	
**[FA 12:2;O - H]-**	MeOH:H_2O	1.27E-04	8.81E-06	
**[FA 12:2;O - H]-**	LLE Lower	0.00E+00	0.00E+00	
**[FA 12:2;O - H]-**	LLE upper	9.14E-04	1.14E-04	
**[FA 12:2;O_2 - H]-**	MeOH:H_2O	2.88E-05	1.51E-05	/2
**[FA 12:2;O_2 - H]-**	LLE Lower	0.00E+00	0.00E+00	/2
**[FA 12:2;O_2 - H]-**	LLE upper	2.53E-03	1.99E-04	/2
**[FA 12:3;O_2 - H]-**	MeOH:H_2O	0.00E+00	0.00E+00	*4
**[FA 12:3;O_2 - H]-**	LLE Lower	0.00E+00	0.00E+00	*4
**[FA 12:3;O_2 - H]-**	LLE upper	1.55E-04	1.05E-05	*4
**[FA 12:4 - H]-**	MeOH:H_2O	0.00E+00	0.00E+00	*4
**[FA 12:4 - H]-**	LLE Lower	0.00E+00	0.00E+00	*4
**[FA 12:4 - H]-**	LLE upper	6.12E-05	7.32E-06	*4
**[FA 12:4;O_3 - H]-**	MeOH:H_2O	0.00E+00	0.00E+00	*4
**[FA 12:4;O_3 - H]-**	LLE Lower	0.00E+00	0.00E+00	*4
**[FA 12:4;O_3 - H]-**	LLE upper	5.30E-05	7.37E-06	*4
**[FA 14:1;O_2 - H]-**	MeOH:H_2O	0.00E+00	0.00E+00	*4
**[FA 14:1;O_2 - H]-**	LLE Lower	0.00E+00	0.00E+00	*4
**[FA 14:1;O_2 - H]-**	LLE upper	2.46E-04	5.70E-05	*4
**[FA 14:1;O_3 - H- H_2O]-**	MeOH:H_2O	0.00E+00	0.00E+00	*4
**[FA 14:1;O_3 - H- H_2O]-**	LLE Lower	0.00E+00	0.00E+00	*4
**[FA 14:1;O_3 - H- H_2O]-**	LLE upper	1.21E-04	5.91E-06	*4
**[FA 14:2 + HCOO]-**	MeOH:H_2O	0.00E+00	0.00E+00	*4
**[FA 14:2 + HCOO]-**	LLE Lower	0.00E+00	0.00E+00	*4
**[FA 14:2 + HCOO]-**	LLE upper	1.40E-04	1.24E-05	*4
**[FA 14:2;O - H]-**	MeOH:H_2O	0.00E+00	0.00E+00	*4
**[FA 14:2;O - H]-**	LLE Lower	0.00E+00	0.00E+00	*4
**[FA 14:2;O - H]-**	LLE upper	6.74E-05	5.07E-06	*4
**[FA 14:4;O + HCOO]-**	MeOH:H_2O	0.00E+00	0.00E+00	*4
**[FA 14:4;O + HCOO]-**	LLE Lower	0.00E+00	0.00E+00	*4

**[FA 14:4;O + HCOO]-**	LLE upper	5.33E-05	1.47E-05	*4
**[FA 16:1;O_2 - H-;]-**	MeOH:H_2O	2.43E-05	1.01E-05	
**[FA 16:1;O_2 - H-;]-**	LLE Lower	0.00E+00	0.00E+00	
**[FA 16:1;O_2 - H-;]-**	LLE upper	6.25E-04	5.82E-05	
**[FA 16:2;O - H]-**	MeOH:H_2O	0.00E+00	0.00E+00	
**[FA 16:2;O - H]-**	LLE Lower	0.00E+00	0.00E+00	
**[FA 16:2;O - H]-**	LLE upper	2.52E-04	2.23E-05	
**[FA 16:2;O_5 - H- H_2O]-**	MeOH:H_2O	0.00E+00	0.00E+00	*4
**[FA 16:2;O_5 - H- H_2O]-**	LLE Lower	0.00E+00	0.00E+00	*4
**[FA 16:2;O_5 - H- H_2O]-**	LLE upper	6.89E-05	1.01E-05	*4
**[FAL 10:1 + HCOO]-**	MeOH:H_2O	3.09E-05	8.35E-06	/2
**[FAL 10:1 + HCOO]-**	LLE Lower	0.00E+00	0.00E+00	/2
**[FAL 10:1 + HCOO]-**	LLE upper	5.18E-04	7.33E-05	/2
**[FAL 10:2 + HCOO]-**	MeOH:H_2O	0.00E+00	0.00E+00	
**[FAL 10:2 + HCOO]-**	LLE Lower	0.00E+00	0.00E+00	
**[FAL 10:2 + HCOO]-**	LLE upper	1.81E-04	1.38E-05	
**[FAL 10:3 + HCOO]-**	MeOH:H_2O	0.00E+00	0.00E+00	*4
**[FAL 10:3 + HCOO]-**	LLE Lower	0.00E+00	0.00E+00	*4
**[FAL 10:3 + HCOO]-**	LLE upper	5.62E-05	1.43E-06	*4
**[FAL 12:1 + HCOO]-**	MeOH:H_2O	0.00E+00	0.00E+00	
**[FAL 12:1 + HCOO]-**	LLE Lower	0.00E+00	0.00E+00	
**[FAL 12:1 + HCOO]-**	LLE upper	2.63E-04	2.96E-05	
**[FAL 16:2 + HCOO]-**	MeOH:H_2O	0.00E+00	0.00E+00	*4
**[FAL 16:2 + HCOO]-**	LLE Lower	0.00E+00	0.00E+00	*4
**[FAL 16:2 + HCOO]-**	LLE upper	4.06E-05	9.23E-06	*4
**[Cer 34:1;O2 + Cl]-**	MeOH:H_2O	0.00E+00	0.00E+00	*4
**[Cer 34:1;O2 + Cl]-**	LLE Lower	0.00E+00	0.00E+00	*4
**[Cer 34:1;O2 + Cl]-**	LLE upper	2.65E-05	1.17E-05	*4
**[MG 18:0 - H- H_2O]-**	MeOH:H_2O	0.00E+00	0.00E+00	
**[MG 18:0 - H- H_2O]-**	LLE Lower	0.00E+00	0.00E+00	
**[MG 18:0 - H- H_2O]-**	LLE upper	4.58E-05	1.50E-05	
**[MG O-12:3 - H]-**	MeOH:H_2O	0.00E+00	0.00E+00	
**[MG O-12:3 - H]-**	LLE Lower	0.00E+00	0.00E+00	
**[MG O-12:3 - H]-**	LLE upper	6.10E-05	1.59E-05	
**[MG O-12:5 + Cl]-**	MeOH:H_2O	0.00E+00	0.00E+00	
**[MG O-12:5 + Cl]-**	LLE Lower	0.00E+00	0.00E+00	
**[MG O-12:5 + Cl]-**	LLE upper	8.17E-05	2.07E-05	
**[LPG 18:1 - H]-**	MeOH:H_2O	0.00E+00	0.00E+00	
**[LPG 18:1 - H]-**	LLE Lower	0.00E+00	0.00E+00	
**[LPG 18:1 - H]-**	LLE upper	2.21E-05	8.58E-06	
**[PE 34:1 - H]-**	MeOH:H_2O	0.00E+00	0.00E+00	
**[PE 34:1 - H]-**	LLE Lower	0.00E+00	0.00E+00	
**[PE 34:1 - H]-**	LLE upper	1.03E-04	2.95E-05	
**[PE 38:3 - H]-**	MeOH:H_2O	0.00E+00	0.00E+00	

**[PE 38:3 - H]-**	LLE Lower	0.00E+00	0.00E+00	
**[PE 38:3 - H]-**	LLE upper	6.60E-05	1.90E-05	
**[PE O-34:2 - H]-**	MeOH:H_2O	0.00E+00	0.00E+00	
**[PE O-34:2 - H]-**	LLE Lower	0.00E+00	0.00E+00	
**[PE O-34:2 - H]-**	LLE upper	8.45E-05	3.37E-05	
**[PE O-36:5 - H]-**	MeOH:H_2O	0.00E+00	0.00E+00	
**[PE O-36:5 - H]-**	LLE Lower	0.00E+00	0.00E+00	
**[PE O-36:5 - H]-**	LLE upper	1.33E-04	5.26E-05	
**[PE O-38:5 - H]-**	MeOH:H_2O	0.00E+00	0.00E+00	
**[PE O-38:5 - H]-**	LLE Lower	0.00E+00	0.00E+00	
**[PE O-38:5 - H]-**	LLE upper	1.28E-04	4.50E-05	
**[PI 34:1 - H]-**	MeOH:H_2O	0.00E+00	0.00E+00	
**[PI 34:1 - H]-**	LLE Lower	0.00E+00	0.00E+00	
**[PI 34:1 - H]-**	LLE upper	1.08E-04	1.69E-05	
**[PI 36:1 - H]-**	MeOH:H_2O	0.00E+00	0.00E+00	
**[PI 36:1 - H]-**	LLE Lower	0.00E+00	0.00E+00	
**[PI 36:1 - H]-**	LLE upper	9.90E-05	1.71E-05	
**[PI 36:2 - H]-**	MeOH:H_2O	0.00E+00	0.00E+00	
**[PI 36:2 - H]-**	LLE Lower	0.00E+00	0.00E+00	
**[PI 36:2 - H]-**	LLE upper	7.78E-05	1.27E-05	
**[PI 38:4 - H]-**	MeOH:H_2O	0.00E+00	0.00E+00	
**[PI 38:4 - H]-**	LLE Lower	0.00E+00	0.00E+00	
**[PI 38:4 - H]-**	LLE upper	2.10E-04	2.24E-05	
**[PS 22:2;O_2 + Cl]-**	MeOH:H_2O	0.00E+00	0.00E+00	
**[PS 22:2;O_2 + Cl]-**	LLE Lower	0.00E+00	0.00E+00	
**[PS 22:2;O_2 + Cl]-**	LLE upper	7.42E-05	2.25E-05	
**[PS 24:2;O_2 + Cl]-**	MeOH:H_2O	1.80E-04	8.20E-05	/5
**[PS 24:2;O_2 + Cl]-**	LLE Lower	0.00E+00	0.00E+00	/5
**[PS 24:2;O_2 + Cl]-**	LLE upper	9.63E-04	2.82E-04	/5
**[PS 36:1 - H]-**	MeOH:H_2O	0.00E+00	0.00E+00	
**[PS 36:1 - H]-**	LLE Lower	0.00E+00	0.00E+00	
**[PS 36:1 - H]-**	LLE upper	9.85E-05	1.54E-05	
**[G6P - H]-**	MeOH:H_2O	1.15E-04	6.04E-05	
**[G6P - H]-**	LLE Lower	7.48E-05	2.53E-05	
**[G6P - H]-**	LLE upper	0.00E+00	0.00E+00	
**[Glycerol 3-phosphate - H]-**	MeOH:H_2O	1.58E-04	4.70E-05	
**[Glycerol 3-phosphate - H]-**	LLE Lower	8.84E-05	1.57E-05	
**[Glycerol 3-phosphate - H]-**	LLE upper	0.00E+00	0.00E+00	
**[GPEA - H]-**	MeOH:H_2O	9.09E-04	1.53E-04	/4
**[GPEA - H]-**	LLE Lower	6.36E-04	1.17E-04	/4
**[GPEA - H]-**	LLE upper	0.00E+00	0.00E+00	/4
**[GPC + HCOO]-**	MeOH:H_2O	0.00E+00	0.00E+00	
**[GPC + HCOO]-**	LLE Lower	7.10E-05	1.20E-05	
**[GPC + HCOO]-**	LLE upper	0.00E+00	0.00E+00	

**[NAT 16:0 - H]-**	MeOH:H_2O	0.00E+00	0.00E+00	
**[NAT 16:0 - H]-**	LLE Lower	0.00E+00	0.00E+00	
**[NAT 16:0 - H]-**	LLE upper	2.16E-04	3.20E-05	
**[NAT 18:0 - H]-**	MeOH:H_2O	0.00E+00	0.00E+00	
**[NAT 18:0 - H]-**	LLE Lower	0.00E+00	0.00E+00	
**[NAT 18:0 - H]-**	LLE upper	1.29E-04	2.13E-05	
**[Taurine - H]-**	MeOH:H_2O	1.09E-02	1.18E-03	/100
**[Taurine - H]-**	LLE Lower	1.07E-02	1.51E-03	/100
**[Taurine - H]-**	LLE upper	1.11E-04	1.68E-05	/100

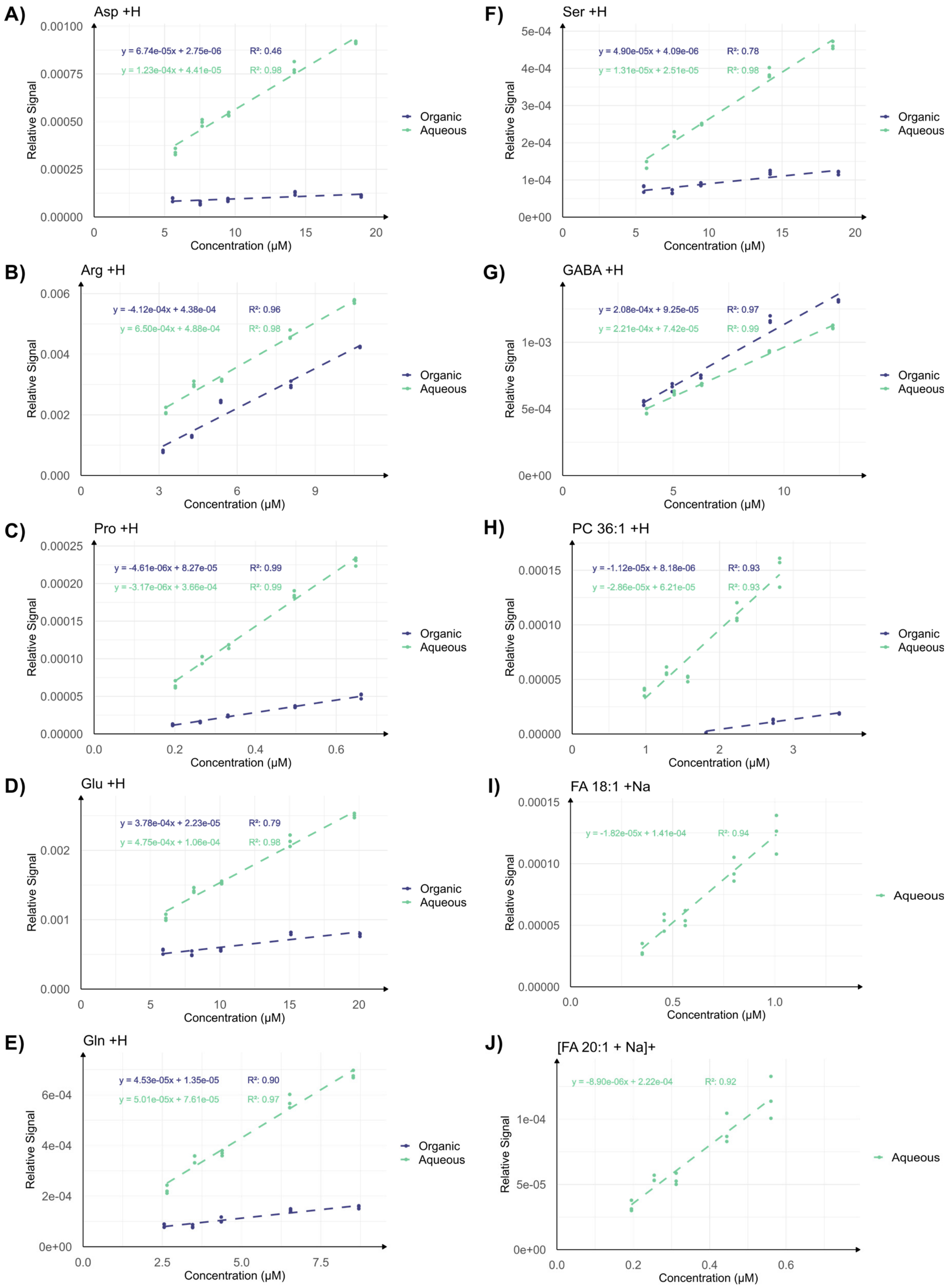

Figure S1. Ionization efficiencies of analytes in solutions containing known concentrations of several molecules. The solutions were prepared in each BUME phase and samples were analyzed using DIP-MS, n=3.

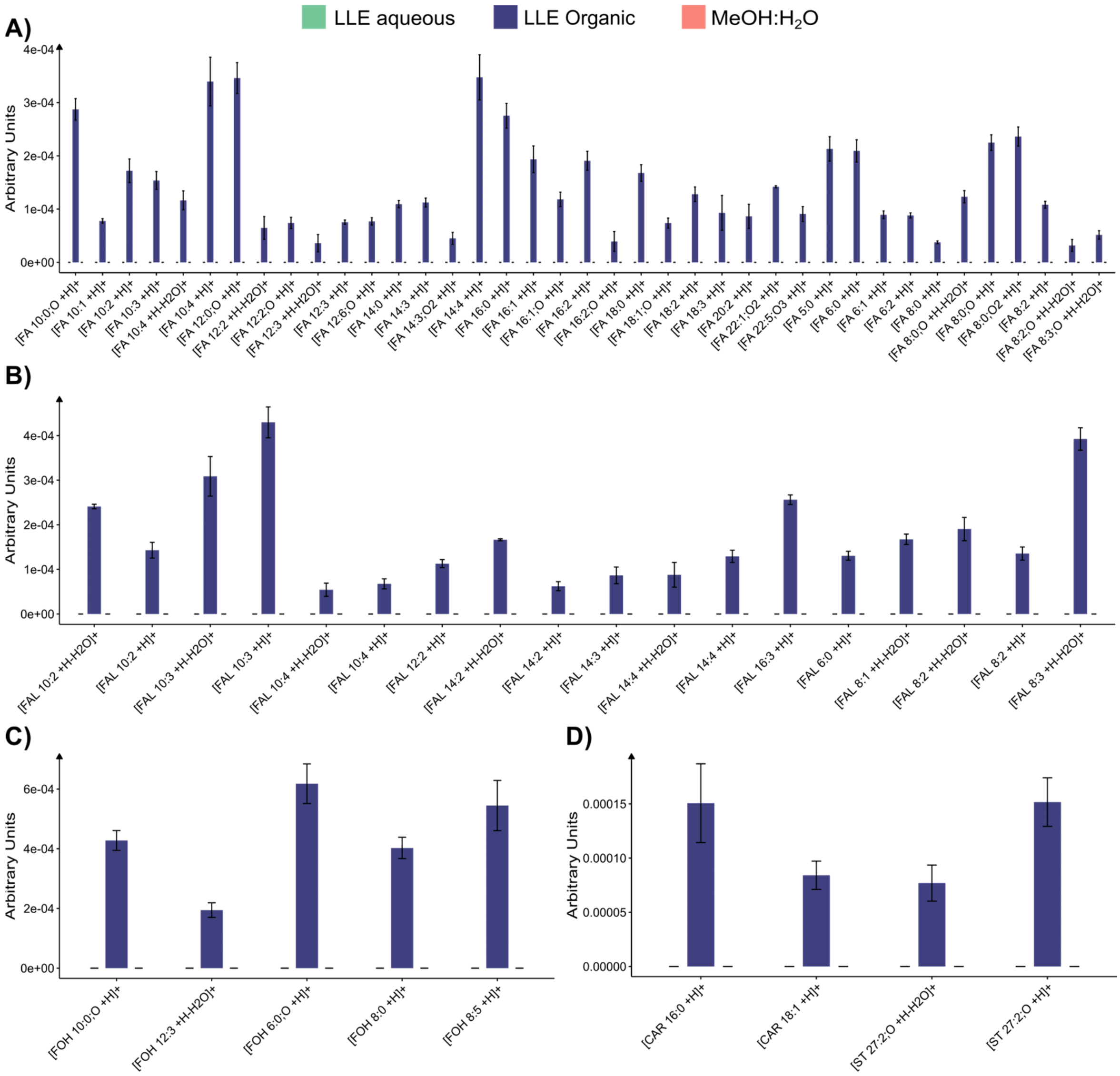

Figure S2. Analytes detected in positive mode from INS-1 cells exposed to 20 mM Glucose for 15 min in the aqueous and organic LLE phases and in MeOH:H_2O. the data from A to D was normalized to TIC. The error bars correspond to one standard deviation of triplicates of sample preparation. To achieve clear visualization of the data, scaling factors were applied hence the need for arbitrary units on the y-axis. Further information can be found in table S7. Abbreviations are fatty acid (FA), fatty aldehyde (FAL), fatty alcohols (FOH), carnitine (Car), steroids (ST).

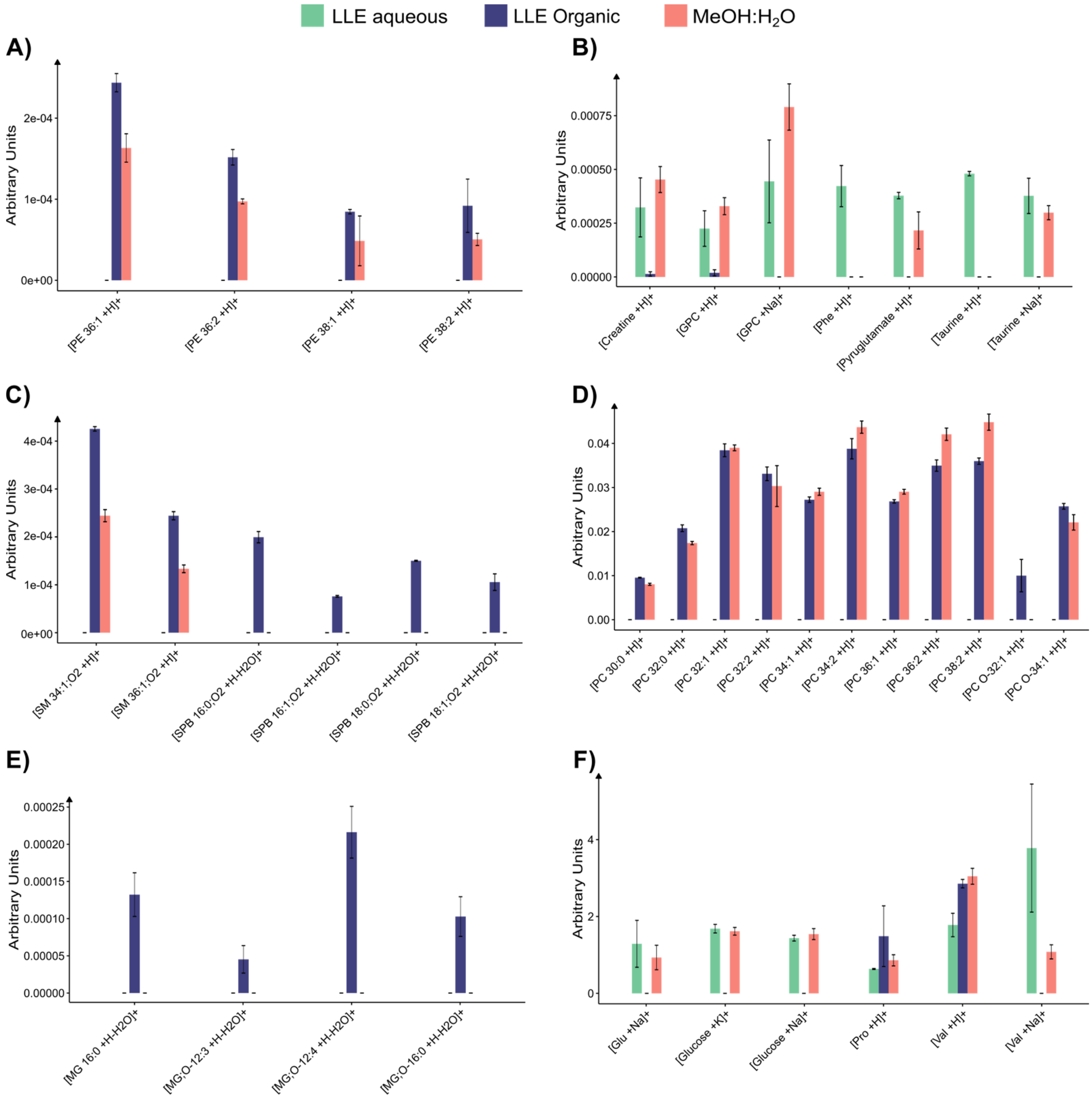

Figure S3. Analytes detected in positive modes from INS-1 cells exposed to 20 mM Glucose for 15 min in the lower and upper LLE phases; and in MeOH:H_2O solvent. These include PE(A),small metabolites (B and F), SM and SPB (C), PC (D) and MG (E). For A, D and F) the signal was normalized to non-endogenous internal standards. The remainder data was normalized to total ion count (TIC). Arbitrary units explained in table S7. The error bars correspond to one standard deviation of triplicates of sample preparation. Abbreviations are PE, phosphatidylethanolamines; SM, sphingomyelins; SPB, sphingoid bases; PC, phosphatidylcholines; MG, monoacylglycerols; GPC, glycerophosphorylcholine.

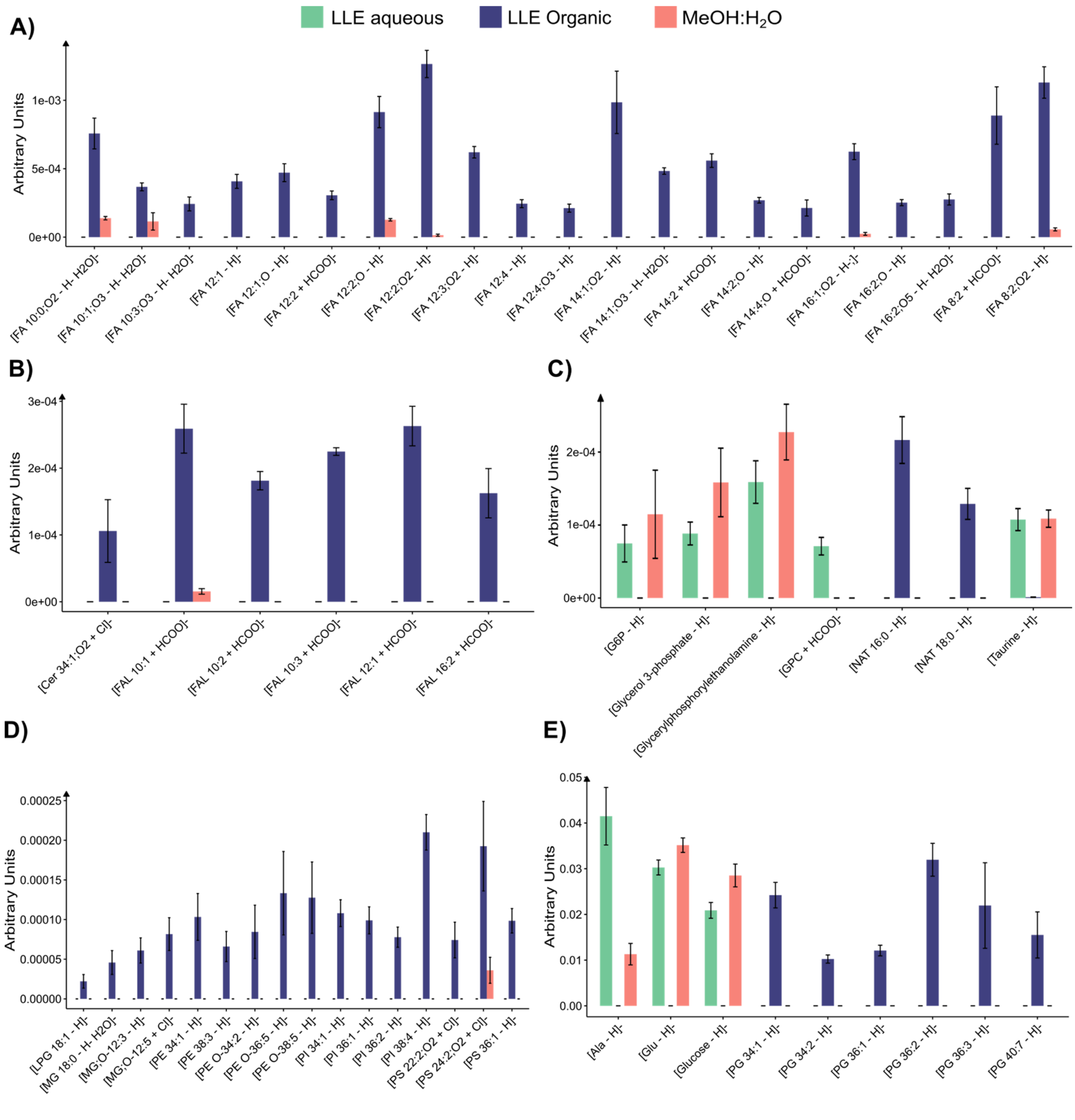

Figure S4. Analytes detected in negative mode from INS-1 cells exposed to 20 mM Glucose for 15 min in the lower and upper LLE phases and in MeOH:H_2O. The analytes in graph A and E were normalized to non-endogenous standards, while the remainder were normalized to TIC. The error bars correspond to one standard deviation of triplicates of sample preparation. To achieve clear visualization of the data, scaling factors were applied hence the need for arbitrary units on the y-axis. Further information can be found in table S8. Abbreviations are FA, fatty acid; FAL, fatty aldehyde, NAT, n-acyl taurines; LPG, lysophosphatidylglycerols; MG, monoacylglycerols; PE, phosphatidylethanolamines; PI, phosphatidylinositols; PS, phosphatidylserines; and GPC, glycerophosphorylcholine.

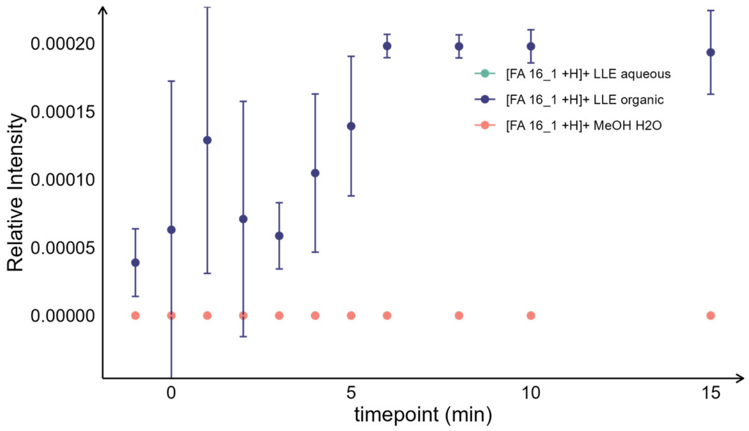

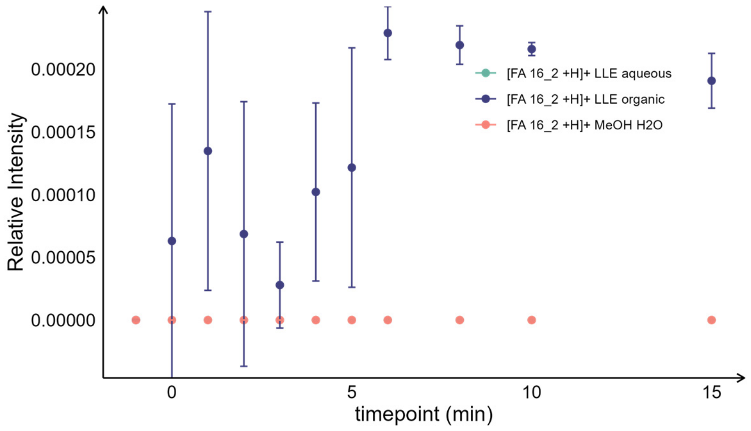

Figure S5. Time resolved information of FA 16:1 (upper) and 16:2 (lower) analyzed with the DIP-MS (orange) and the aqueous (green) and organic (purple) phases of the online LLE DIP-MS from INS-1 cells exposed to 20 mM glucose for different times (0, 1, 2, 3, 4, 5, 6, 8, 10, 15 min). The signals have been normalized to TIC. The error bars correspond to one standard deviation of triplicates of sample preparation.
